# On Power Line Positioning Systems

**DOI:** 10.3390/s22207827

**Published:** 2022-10-14

**Authors:** Lisandro Lovisolo, Fernando Cruz-Roldán, Manuel Blanco-Velasco

**Affiliations:** 1 Department of Electronics and Communications Engineering, Rio de Janeiro State University (UERJ), Rio de Janeiro 999074, Brazil; 2 Department of Signal Theory and Communications, University of Alcalá (UAH), 28871 Alcalá de Henares, Spain

**Keywords:** positioning, power line positioning system, power line communications

## Abstract

Power line infrastructure is available almost everywhere. Positioning systems aim to estimate where a device or target is. Consequently, there may be an opportunity to use power lines for positioning purposes. This survey article reports the different efforts, working principles, and possibilities for implementing positioning systems relying on power line infrastructure for power line positioning systems (PLPS). Since Power Line Communication (PLC) systems of different characteristics have been deployed to provide communication services using the existing mains, we also address how PLC systems may be employed to build positioning systems. Although some efforts exist, PLPS are still prospective and thus open to research and development, and we try to indicate the possible directions and potential applications for PLPS.

## 1. Introduction

Positioning systems aim to estimate where a target is. Meanwhile, power line infrastructure is available almost everywhere. Consequently, it may be advisable to use power lines for some applications requiring positioning estimates in some scenarios and environments. This work reports how power lines and Power Lines Communication (PLC) systems may be employed opportunistically to design systems capable of returning position estimates.

Positioning applications include the localization and tracking of persons and lost objects or assets in industrial buildings as well as context- and position-dependent data forwarding as in museums and guided tours, to name a few [1,2,3]. The required positioning accuracy is highly dependent on the application and technology, since the position estimate can be a region the target is, its locality, its coordinates, or the relative distance to a reference (ranging from meters to millimeters). Positioning systems have been designed aiming at global coverage, the so-called Global Positioning Systems (GPS), and to operate inside buildings, the so-called Indoor Positioning System (IPS). Although in outdoor scenarios, GPS technologies are the de facto standard, technology development for cost-effective IPS is still ongoing [1,2,3,4]. Different technologies and strategies have been employed. The target device (or tag) to be located normally collects data from anchors to resolve the positioning; however, systems that collect data from the target or its effects also exist. Some positioning systems opportunistically employ references from legacy systems—as mobile communication (GSM, 3G, 4G), local and personal area networks (WiFi, Bluetooth, UWB), and Radio-Frequency Identification technologies, or also from emerging communication systems such as Visible Light Communication (VLC), to name a few—to resolve the position.

**Figure 1:**
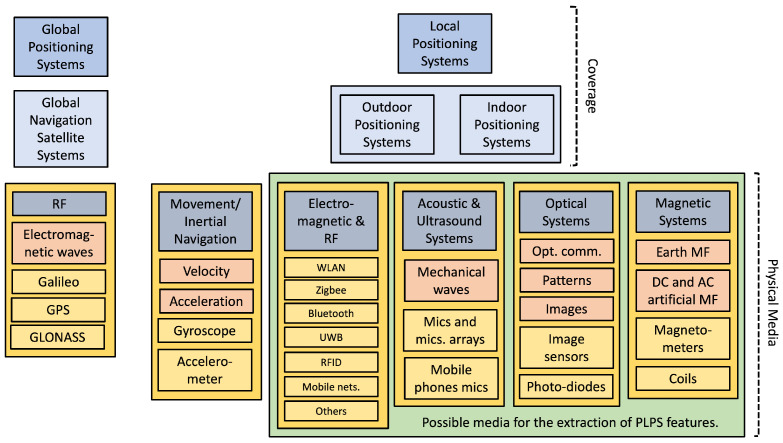
Schematic view of systems and physical quantities used for positioning. Some systems are specially designed for positioning, such as the GPS, while others opportunistically employ legacy systems for positioning. The latter may rely on artificial or nature-born information. The green rectangle indicates the possibilities for PLPS construction—the characteristics, technologies, and applications for PLPS are the subject of this work.

Positioning systems employ different physical quantities and techniques within different coverage areas [1,2,3,5,6,7,8,9,10,11,12,13,14,15,16,17,18,19,20,21,22,23], as shown in Figure 1. In the figure, we separate the positioning systems into global and local. The global ones are designed to operate almost worldwide. Meanwhile, the local ones return a relative position to a local reference, since the operating principles and their interplay with constructions and other objects generally lead to different constraints that restrict their usage.

Figure 1 also presents (though not exhaustively) the different physical principles and quantities employed and the corresponding sensors for collecting the positioning features. Some features are artificial, while others are nature-born. In this work, we discuss the possibilities of obtaining positioning features from the power line infrastructure.

We borrow the name power line positioning system (PLPS) from [24] but expand it to include any system relying on the electrical wiring, the environmental effects of the energy and signals it may conduct, or the effects of persons on signals flowing in the lines to obtain position estimates. PLPS might be easier to deploy and are less visible than radio-frequency (RF) counterparts. Wiring placement is more stable over time than RF access points disposition; consequently, the databases used in some PLPS may be up to date longer than those built for RF signals-based positioning. Complementary, Power Line Communication (PLC) systems [25] are being deployed to support different applications in several scenarios, presenting an opportunity for designing PLC-based IPS (PLC-IPS).

There are many tutorials, surveys, reviews, and books on positioning systems [1,5,6,13,14,17,26,27] and on the use of electrical wiring for communications [25,28,29,30]. Nevertheless, they do not delve into using power lines for positioning. This article’s main contribution is presenting how the power line infrastructure can be used for positioning. We survey the different efforts, working principles, implementation possibilities, and application scenarios that could benefit from PLPS and PLC-IPS. We analyze the positioning references that can be derived from or inserted in the power lines. We provide a systemic categorization of PLPS based on the interaction between the target and the remaining components of the positioning system. In this sense, the use of positioning infrastructure equipment or a target that helps for resolving the position is discussed. For each category, we summarize existing efforts, the possible features for computing the positioning, the techniques to acquire them, and the methods to resolve the target position.

Section 2 discusses general aspects of PLPS and introduces some classification dimensions. The systematic analysis of the use of the electrical wiring, mains, PLC, and the system characteristics, operation principles, and configurations for positioning lead to three eclectic PLPS categories. The Infra-less Target-based PLPS are presented in Section 3. The target employs the mains for self-positioning directly without interaction or injection of positioning reference signals (beacon) in the mains, i.e., without any specific PLPS infrastructure. The next broad category is the Infra-based Target-obtuse PLPS presented in Section 4. The power lines are used to deploy unobtrusive positioning systems deploying the adequate PLPS infrastructure without any support from the target. Then, Section 5 discusses the general class of Infra-based Target-based PLPS. It rests on an infrastructure to inject positioning beacons into the mains that are irradiated or relayed for the target to extract features for positioning. Despite the many positioning technologies, some applications and operation scenarios impose defying aspects that may require new positioning technologies. We discuss the problems in each system category and how PLPS can be used in real scenarios in Section 6. Section 7 concludes this survey and prospective article on the use of power lines and power line communications for positioning.

## 2. Power Line Positioning Systems: Characteristics, Properties and Categories

To the extent of our knowledge, the use of the electrical wiring to irradiate specific positioning purpose signals can be traced to two predecessors [24,31]. In [31], the power lines irradiate a synchronization beacon for an acoustic positioning system. Differently, in [24], the power lines are directly used to irradiate tones that are received using a loop antenna, and the sensed tones’ amplitudes are taken as features for fingerprinting positioning.

Autonomous robots may plug into standard, unmodified electrical outlets for recharging, extending the robot autonomy and displacement range, and dispensing particular bases/outlets. The robot must master several movements for plugging in usual outlets, but first off, it must search and find the power outlet. Vision can be used to identify the outlets. However, alternative approaches may rely on sensing the Electromagnetic Field (EMF) generated by the AC fundamental frequency to detect and align to the outlet [32]. Similarly, wireless power transfer maximization depends on correct alignment [33,34].

Using power lines for positioning in an obtrusive manner, more recently, ref. [35] utilizes the effects of electromagnetic coupling on a wave propagating through the electric wiring for occupancy detection (pinpoint the room region a person is from the effects of its body).

The monograph [6] classifies power line-based IPS within “Infrastructure Positioning Systems” utilizing the existing building infrastructure directly or embedding the IPS into the building, hiding the system from its users. Therefore, since a PLPS benefits from the installed wiring, it is infrastructure-dependent. IPS technologies may be building dependent or independent [27]. Building independence refers to disregarding any particular hardware or knowledge of the building for the IPS to work. If building-dependent, the technology employs prior knowledge of the building or some technology existing inside it for resolving the position.

To analyze the different characteristics and possibilities of PLPS, we need to analyze relevant system properties: (i) the introduction of positioning beacons and, subsidiary, the infrastructure that introduces them to make the PLSP operational; (ii) the measurements leading to the positioning features and who accomplishes them; (iii) if the computation of the position estimate is performed at the target or the infra and if there is a collaboration between the target and the PLPS infra to resolve the position. Although some of these characteristics are not exclusive, they can be used to define the three PLPS categories in Table 1.

### 2.1. Positioning Reference Signals

If the PLPS employs reference signals irradiated either intentionally or not by the mains for positioning purposes, we refer to it as Infra-based. The injection of positioning beacons in the mains requires specific drivers and coupling devices, which is an infra for the PLPS. Alternatively, the PLPS may rest on seeing the parts of the mains or sensing the effects of the energized power lines without using a positioning-purpose reference signal. Consequently, an Infra-less (system) refers to PLPS relying solely on the electrical wiring and its parts.

Due to the increasing use of PLC, it appears as a seamless candidate for providing positioning references. Thus, PLC-IPS refers to PLPS utilizing the PLC signals for an IPS. Nevertheless, the large-wavelength and low-intensity EMF is challenging to detect; thus, alternatively, PLC to wireless (PLC/RF) or PLC to light (PLC/VLC) hybrid links may help [36,37,38,39,40,41]. The anchor emitting the reference signal is connected to the outlet or the light socket to up-convert and irradiate the positioning signal conveyed by the PLC; the position computation may rest on many different methods used by wireless- and light-based positioning. Similarly, some PLC subcarriers can excite the magnets for magnetic-ranging positioning.

### 2.2. Collecting the Features

In some PLPS, the target can collect the positioning features from the reference signals, the environment, or other contextual data to compute its position. In this case, we say that the PLPS is Target-based. Otherwise, if the PLPS infrastructure acquires the positioning features, we say it is Target-obtuse.

### 2.3. Computing the Position Estimate

The position estimate can be determined either at the target or infra. Notwithstanding, it is common to envision cooperative Infra-based Target-assisted (Infra-Target cooperation) approaches when the target collects the features (Target-based) and sends them to a positioning server where the target position is estimated. In the case that the position estimate is computed by the target without specific PLPS infrastructure, we say it is an autonomous or self-positioning system. On the other hand, if the PLPS infrastructure computes the target position without target support, we say the system is unobtrusive.

### 2.4. System Categories

Using the discussed properties, three different PLPS categories follow, which are shown in the first column of Table 1. In Infra-less Target-based (ILTB) PLPS architectures, discussed in more detail in Section 3, the target employs the mains without collaboration or support from other infrastructure to resolve the position. Thus, we refer to this paradigm as autonomous or self-positioning.

The Infra-based Target-obtuse (IBTO) PLPS architecture is discussed in more detail in Section 4. It resolves the target position from the target effects on position-purpose reference signals flowing through the wires. If the target is unaware of the PLPS, we say the PLPS is unobtrusive.

The Infra-based Target-based (IBTB) PLPS architecture is discussed in more detail in Section 5. The electrical cables are used to irradiate positioning beacons or transport them for irradiation by an auxiliary infrastructure. The target acquires the positioning features from the reference signals. Any IBTB PLPS is transparent, since the target is aware of the PLPS infrastructure.

### 2.5. PLPS Examples

Table 2 lists the different systems and experiments regarding PLPS design. The works reported in Table 2 indicate possible positioning features from power lines collected using different techniques for different perspectives. The first column refers to the purpose of the positioning system (its application or service) presented in the reference in the last column. The second column maps the work in the three PLPS categories previously presented. Works that are not explicitly PLPS related are indicated using “none”; they are included in the table, since they present experiments and results that may push forward the design and deployment of PLPS. The third column describes the originating physical principle for the feature. The fourth column presents the feature source, which is followed by how it is collected. The sixth column reports the measurement distance (reach) for feature collection, and the seventh presents how the feature is employed for obtaining the position estimate. The eighth column links the feature bandwidth to the working bands of the PLC standards (see Appendix A).

In the following Section 3, Section 4 and Section 5, we discuss the role of the power line infrastructure in different power line positioning technologies. We present examples of systems in each category. We discuss the positioning references or features that can be employed. Part of the features is naturally provided by the power lines; this can lead to infra-less positioning systems. Meanwhile, infra-based PLPS intentionally insert signals in the power lines to support positioning. For the three system categories, we also discuss how to collect the positioning features and possible approaches to resolving the target position.

## 3. Infra-Less Target-Based (Self) PLPS

**Figure 2:**
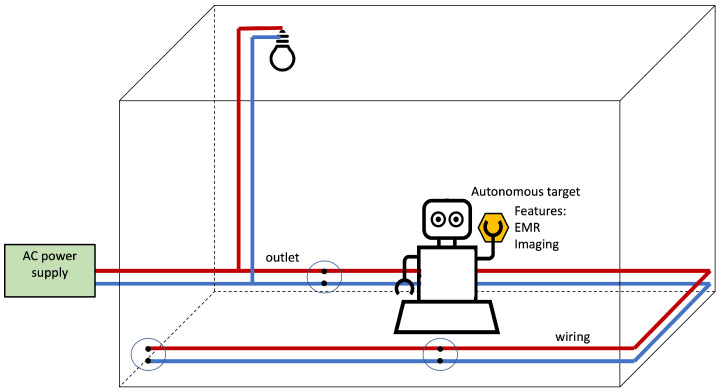
Infra-less Target-based PLPS architecture. The PLPS components are highlighted in yellow. In this scenario, the target uses the electrical network for positioning without PLPS infrastructure.

Figure 2 illustrates the Infra-less Target-based PLPS architecture. The autonomous device senses the environment searching for position clues related to the electrical wiring for self-positioning. The features can be any information related to the power system. They derive from the mains infrastructure, the irradiated EMF, or images to, for example, recognize the outlets or the light fixtures (although if the light conveys positioning-oriented data, we refer to the PLPS as Infra-based).

### 3.1. Examples

Let us discuss the operation principles of the ILTB PLPS in rows two to seven in Table 2 [32,42,43,44,45].

#### 3.1.1. Autonomous Plugging-In

Autonomous plugging-in requires accurate alignment (relative positioning) between the plug prongs and the outlet socket holes with millimeter-scale precision. In [42], the electric field generated by the outlet is used to sense it. Once an outlet is detected, the robot switches to a vision system for plugging in. In [32], the AC carrier effects guide plug insertion utilizing two separate electrodes, subsequently. First off, a disc of copper foil on the plug front senses the electric field to support coarse alignment with the outlet at mid-range. At a closer range, the ground prong (of a smaller cross-sectional area than the copper foil) is used to measure the electric field for plugging in. A scanning procedure that maximizes the field strength is employed separately in both the vertical and horizontal directions for alignment. These systems’ characteristics are listed in rows two to five of Table 2.

#### 3.1.2. Autonomous Pearching

Self-positioning for Unmanned Aerial Vehicle (UAV) perching to power lines may provide ubiquitous recharging power sources and extend the autonomy of an UAV [43,44]. Such initiatives are listed in the sixth row of Table 2. The authors in [43,44] claim that the near magnetic field of transmission lines in the medium voltage section can provide sufficiently accurate and low latency measurements for the UAV to estimate its relative position and pearch. Similar concepts may apply to unmanned vehicles in multistory parking garages using magnetic field measurements [55].

#### 3.1.3. AC EMR Fingerprinting Positioning

Inside buildings, steel and reinforced concrete structures and the different electric sources produce both static and extremely low-frequency magnetic fields [56]. If the magnetic field pattern inside a building has sufficient local variability and stability over time, one can use it for fingerprinting positioning [56,57,58]. This approach does not require deploying particular infrastructure, and three-axis magnetometers, such as those embarked in mobile phones for geomagnetic sensors, can cope with the measures necessary to collect the position-dependent features.

### 3.2. Positioning Features

**Figure 3:**
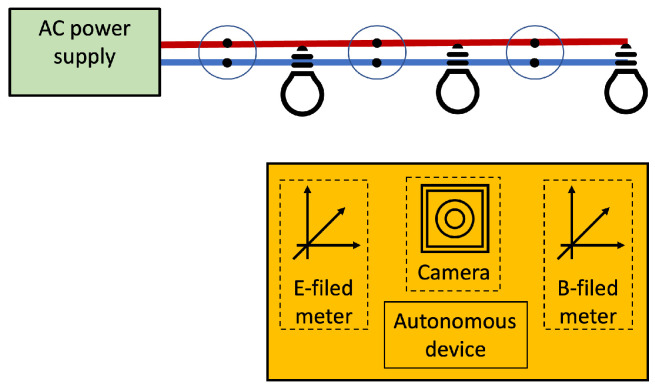
Sensors employed for self-positioning by some Infra-less Target-based PLPS.

The device performing the self-positioning uses clues naturally provided by the power lines for positioning. Figure 3 illustrates the possibilities. Image and video processing techniques can be employed to detect and recognize outlets and lighting fixtures. Magnetic and electric fields are also naturally produced by the AC mains; although, the very low frequency of the mains hardens the detection.

### 3.3. Sensors to Embark

Electronic systems suffer the interference of the AC fundamental; the electric network frequency appears in audio and electrocardiogram recordings [59,60], to place a few examples. The ILTB self-positioning PLPS can use this phenomenon. The target can employ magnetic- or electric-field sensors to perceive the AC EMR [45,61,62]. A microphone, a boosting tank circuit, conductors (measuring in centimeters), and small mics (as the ones embarked on mobile phones) can be used to sense the fundamental EMR and capture the mains’ hums. Many specialized sensors have been developed to sense magnetic fields [63]. As discussed, the target may embark image sensors.

### 3.4. Resolving the Position

As above exemplified, an ILTB PLPS is restricted to using positioning features resulting from the AC EMR amplitude pattern in space, besides imaging.

The intensity of the magnetic field produced by a three-phase single circuit power line is derived in [64]. For a power line consisting of three conductors lying on the plane with an inter-distance of *s* meters (the cables are placed at −s, 0, and *s* on the plane axis perpendicular to the power lines cross-sections), conducting currents *I* (modulus of the phases with angles $-\frac{2\pi }{3}$, 0, and $\frac{2\pi }{3}$), the intensity of the magnetic field at the distance *d* in the direction $\psi_{d}$ (considering the line crossing the three conductors perpendicularly) is

(1) $$\begin{array}{c} B\left(d\right)=\frac{\mu_{0}Is}{2\pi d}{\left(\frac{3d^{2}+s^{2}}{d^{4}-2d^{2}s^{2}\cos\left(2\psi_{d}\right)+s^{4}}\right)}^{\frac{1}{2}}. \end{array}$$

By measuring *B*, one can estimate the distance *d* to the power line for the UAV to pearch for recharging.

Image processing and vision techniques on the visible and non-visible bands of the spectra have presented an astonishing advance in the last decade. These techniques can be used to recognize and extract the distance and relative position from many parts of the power system.

The long wavelength of the EMR resulting from the AC fundamental frequency and its, in general, weak intensity indoors may hamper accurate positioning. However, the authors in [45] report that the spatial variation and temporal stability of the mains EMR promisingly hold up for sub-meter accurate positioning in real time. Fingerprinting positioning methods, also called database correlation or scene analysis, employ position-dependent features, i.e., a database of reference patterns with the associated positions [26]. The target collects a fingerprint that is compared to the reference ones to estimate the target position. This strategy can be employed to use the spatial variation and temporal stability of the AC EMR for resolving the target position.

**Figure 4:**
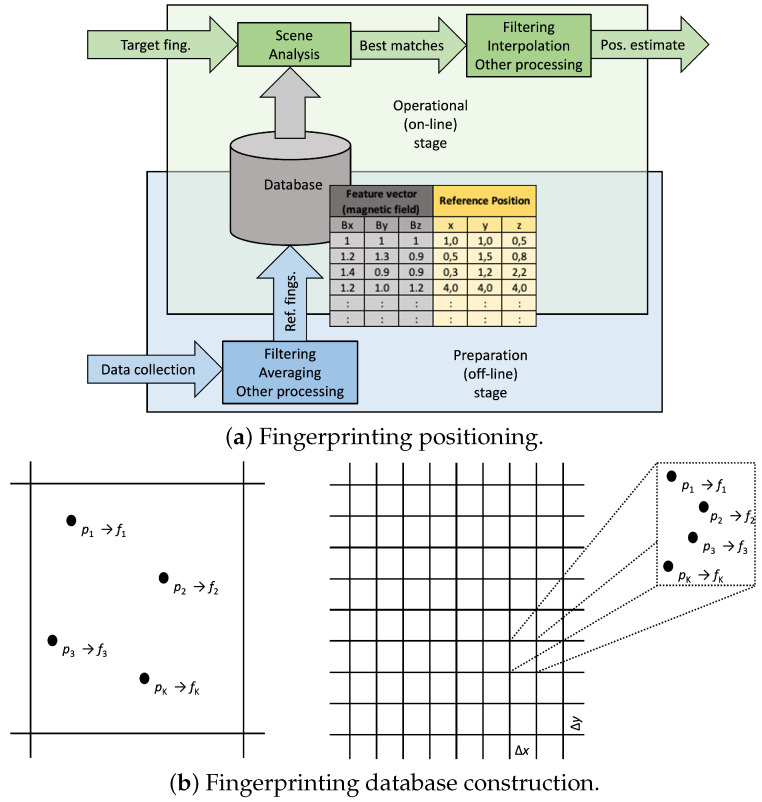
Fingerprinting positioning consists of two stages. During preparation, the database is collected. The feature vector comprises the AC EMR in each axis. In the operational phase, the scene analysis algorithm returns position candidates for the target position. At the bottom, one sees that a fingerprint may contain the positioning features collected at one position (left) or combine the features collected within a cell.

Figure 4a illustrates how fingerprinting works [26]. During operation, the target fingerprint is collected, and the scene analysis algorithm returns position candidates by comparing the target $f_{\mathrm{target}}$ to the reference $f_{k}$ fingerprints. A feature vector $f_{k}$ is collected at position $p_{k}$, *K*; such tandems compose the database of fingerprints, as illustrated at the left of Figure 4b. The scene analysis returns the estimates position

(2) $$\begin{array}{c} {\hat{p}}_{\mathrm{target}}=p_{k}\mathrm{with}k=\arg_{k=\ldots K}\max\mathrm{similarity}\left(f_{\mathrm{target}},f_{k}\right). \end{array}$$

There are many different algorithms for making this comparison and obtaining position candidates, and one may select the position associated with the best match among the reference fingerprints or combine (average/interpolate) the reference position of ranked matches.

Figure 4b depicts the database construction (preparation or off-line) stage when the reference fingerprints, the feature vectors, are collected at known positions. It is common to divide the operating region into cells of size Δx×Δy, associating a fingerprint to one cell—each cell corresponds to a small locality. For the fingerprint, one makes different measurements to confront the inherent variability and stores a few examples, their average over space and time, or the empirical distribution (or parameters modeling it). The overlap of the two stages in Figure 4a indicates that the database can be updated during operation to improve the system’s accuracy and reliability if the working environment requires keeping the databases up to date and valid to work properly.

The authors in [45] present an akin approach, capturing the AC fundamental EMR and inputting it into a fusion-based Simultaneous Localization and Mapping (SLaM) system. Line seven of Table 2 lists this system’s characteristics. The power network EMR pattern across space is combined with a dead-reckoning module. (Velocity is the rate of change in spatial position, and acceleration is the rate of velocity change. From the velocity, one may compute the position change/displacement, and from the acceleration, one may compute the velocity. Movement and inertial navigation systems compute the displacement using the laws of motion [7]. Nowadays, micro-electromechanical systems are embarked on different objects to measure the movement-related physical quantities used to estimate the trajectory and give the relative displacement to the trajectory starting point. These systems may also employ a compass as a complementary datum. Such methods are generally called dead reckoning: the device determines its current position from the past one and its trajectory.) To compute the route, ref. [45] uses a dead-reckoning module, but it employs the received AC fundamental EMR to detect loop closures in the route, in a fingerprinting fashion, recognizing if a point is being revisited.

The ILTB PLPS operational principles discussed in this section rely on sensing the AC mains effects or “seeing” the mains infrastructure. The target is responsible for acquiring the features and computing the position estimate without any additional PLPS infrastructure; besides, the power system is unaware of generating the positioning references. However, it is possible to foresee that there may be data exchange with a server (sending the measurements or querying databases) for the position computation.

### 3.5. Applications

The ILTB self-positioning PLPS may extend the autonomy of robots and electric vehicles. It seems reasonable to include this ability with the many other capabilities envisioned for these machines. Other appliances, such as autonomous vacuum cleaners, could also benefit from this ability. The same concept applies to different machines used in industries and underground mines, to name a few other application domains.

## 4. Infra-Based Target-Obtuse (Unobtrusive) PLPS

**Figure 5:**
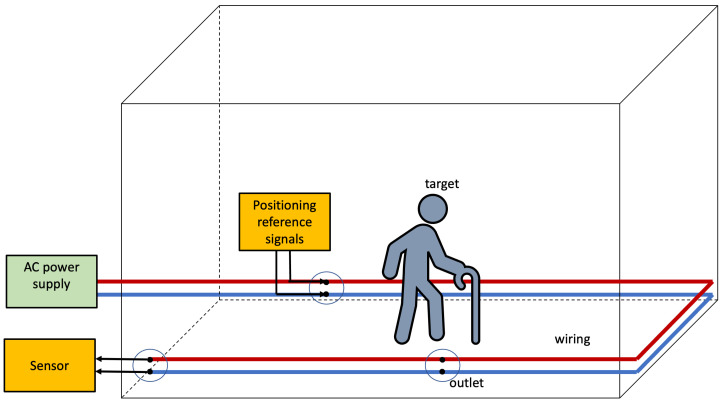
Infra-based and Target-obtuse unobtrusive PLPS architecture. The PLPS components are highlighted in yellow. In this scenario, the system estimates the position of the target whilst the target is unaware.

Figure 5 depicts the basic architecture for the Infra-based Target-obtuse PLPS discussed in this section. The PLPS infrastructure injects signals into the wiring and collects the features at different points. The electromagnetic coupling between the human body and the wiring affects wave propagation in the wires. The wiring and the coupling between the lines and the Earth change much slower than a person moves. Consequently, the PLPS rests on detecting coupling effects changes that are most likely due to humans. Notice that the person might be unaware of the IBTO PLPS; i.e., it may unobtrusive. Such systems may be employed for simple detection to indicate if a room or locality is occupied.

### 4.1. An Example

The IBTO PLPS is illustrated by the example in the eighth row in Table 2. The work [35] injects a 70 MHz reference into the neutral wire and measures its peak value at five outlets. These features are used to detect and locate the person using a support vector machine. The classifier is trained using features collected with the person positioned among 1.2 m × 1.2 m squares in a 9.6 m^2^ room, achieving an accuracy of 91% accuracy and the authors report that mis-classification increased the closer the person was to the near-field region and that tiny movements of the occupants led to significant changes in the sensed tone peak value. Since only persons are expected to move within short periods, filtering out ambient factors (furniture, equipment, vibration, climate) that can make the mean of the peak value wander over long-term measurements may improve performance [46].

### 4.2. System Model

The IBTO PLPS rests on injecting reference signals into the electrical wiring and identifying the distortions they suffer due to the effects on human bodies. In addition to the injection of the reference signals, the IBTO PLPS infrastructure comprises sensors to measure, collect the features and communicate them. We note that PLC technology could support that.

**Figure 6:**
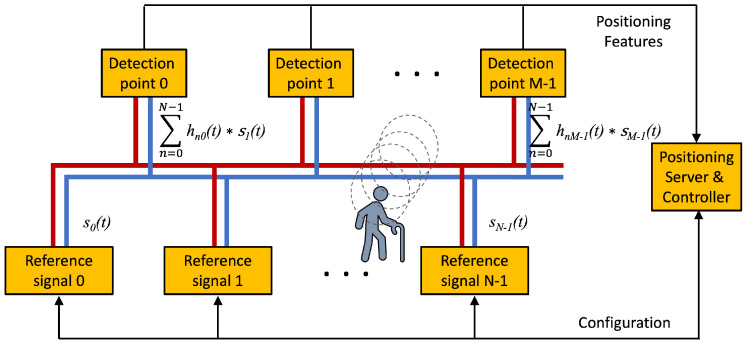
Basic scheme for Infra-based Target-obtuse PLPS.

Figure 6 presents a simplified model for IBTO systems. *N* reference signals are injected at *N* different points. They arrive at *M* detection points. If $h_{nm}$ is the channel response from the *n* reference-signal insertion point to the *m*th detection point, one has

(3) $$r_{m}\left(t\right)=\sum_{n=0}^{N-1}h_{nm}\left(t\right)*s_{n}\left(t\right),$$

where there are NM channels. In the diagram in Figure 6, the references are forwarded to the Positioning Server to resolve the position of the person. The reference signals can be configured by the Controller which decides their frequencies, modulations, and time-division multiplexing.

#### Using PLC for IBTO PLPS

The above model is valid for PLC signals. The simplified model for a PLC symbol is

(4) $$s\left(t\right)=\sum_{c=0}^{C-1}a_{c}e^{j\frac{c2\pi }{C}},$$

where *C* is the number of subcarriers, *c* is the subcarrier index, and $a_{c}\in C$ is the symbol conveyed by the *c*th subcarrier and depends on the constellation applied to modulate it. The Appendix A contains a few details. Thus, using subcarriers from the PLC-OFDM symbol of appropriate frequencies, the changes in the channels due to the coupling between the lines and persons moving in the target area can be detected.

### 4.3. Positioning Features

From the reference signals, one extracts the positioning features to resolve the positioning. They are expected to depend on the person. If one assumes that one can separate the sources, which can be achieved by time or frequency multiplexing, the set of positioning features for the person at a position *k* is given by

(5) $$\begin{array}{c} f_{k}={\left\{\mathrm{features}(h_{nmk}\left(t\right))\right\}}_{n=0\ldots N-1,\;m=0\ldots M-1}. \end{array}$$

If each reference signal is designed to provide *P* features, then at best, there are NMP positioning features, thus

(6) $$\begin{array}{c} f_{k}={\left\{{\left[f_{nmk}^{p}\right]}_{p=0\ldots P-1}\right\}}_{n=0\ldots N-1,\;m=0\ldots M-1}, \end{array}$$

where ${\left[f_{nmk}^{p}\right]}_{p=0\ldots P-1}$ is a vector of features $f_{nmk}^{p}$ derived from $h_{nmk}\left(t\right)$.

Note that simple features can be derived using a set of carriers in each injection point and measuring their amplitudes at the different sensing points. In principle, more carriers increase the capacity to detect the person and discriminate where she is. The insertion points for the tones and their injection powers affect the cover of the IBTO PLPS and its operational area in a given building. The more carriers and injection and sensing points, the more resilient the IBTO is expected to be due to the possible diversity gain.

### 4.4. Resolving the Position

One may apply anomaly/change detection techniques to the features for alarming the presence or movement of a person.

If finer locality information is desired, it is perhaps necessary to acquire prior information about the operating environment and the effects of persons on it, i.e., utilize fingerprinting. An offline preparation phase must occur to learn the signal features with and without the person coupling effects at different positions. So that, after, during operation, they can be associated with different positions.

### 4.5. Applications

The PLPS discussed in this section rests on the coupling among humans and wires to detect the person’s presence and provide her locality indication.

The ubiquity of wireless networks led to the concept of passive positioning systems [65]. The active systems employ tags/electronic devices in the target to be tracked [66] that assist in resolving the position—they are Target-based. In opposition, sensors are deployed in passive systems to capture the possible effects of the target presence. Such systems have been used to detect people [65,67], to resolve their localities (and ultimately the positions) [65,68,69,70], and to assess the individuals in a given region [68]. Such human-presence detection systems are device-free because the target does not carry a sensor. The device-free system is unobtrusive, since it does not disturb the persons being located that could even be unaware of it. As we have seen, the IBTO PLPS may passively detect a person and estimate her locality.

**Figure 7:**
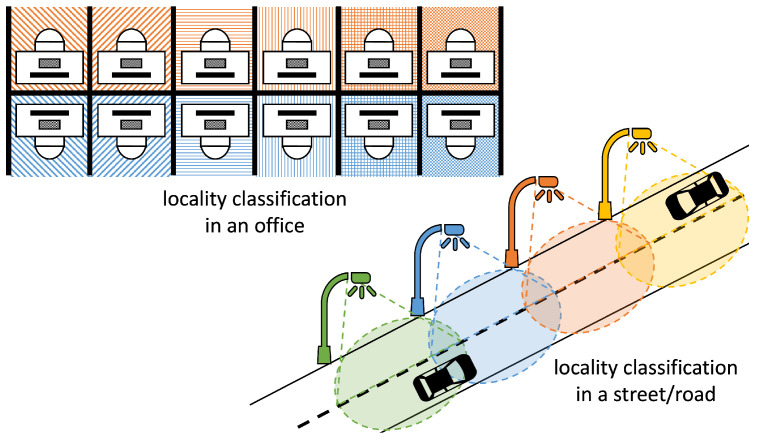
Examples of locality classification systems.

Locality Classification (LC) answers if a target (object or person) is within a given site [71]; if it is inside/outside a region, and, in some cases, the area in the region where it is. Figure 7 illustrates the concept. The upper-left corner illustrates LC in an office. The locality may range from the coarser indication of the row of booths (top or bottom, as illustrated by the colors), the column (the different patterns), and the finer indication of the booth (the blending of colors and patterns). The down-right corner illustrates the LC of road segments according to the light pole providing the illumination (as indicated by the different colors). The size of the regions depends on the number of references, their powers, and the propagation environment. Although LC may present a small resolution, it is expected to present less error than reporting coordinate estimates.

Considering the appropriate constraints, the IBTB PLPS that aims for presence detection and person LC can operate in office and residential buildings, houses, hospitals, nursing homes, and other facilities. It can be used for detection, alarming, or pinpointing the presence of persons (interns, patients, and elders) in a given room to reduce hazards [67,72,73,74]. Such cognizance of the building usage and occupancy helps to understand and manage energy usage [20] and provides context information for controlling electric appliances in smart homes [75,76,77]. Although it employs a specific infrastructure, it can operate independently of the target knowledge of its existence and people’s agreement and be unobtrusive. Consequently, IBTO PLPS also enables intruder detection, substituting many acoustic (ultra-sound) devices and infrared detectors and cameras deployed to detect people’s presence in rooms.

## 5. Infra-Based Target-Based PLPS

**Figure 8:**
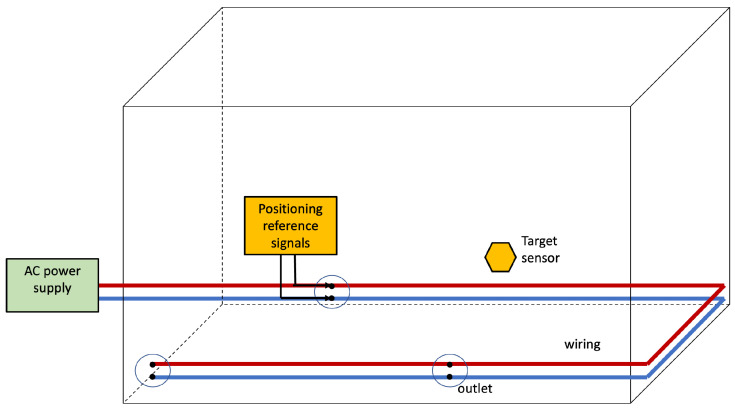
Infra-based and Target-based PLPS architecture. The PLPS components are highlighted in yellow. In this scenario, the electrical network and the target collaborate to obtain the position estimate.

Figure 8 depicts the basic architecture for Infra-based Target-based PLPS. The target senses the positioning reference signals injected into the power line by the IBTB PLPS infrastructure. The target collects the features that are used to estimate its position. The target can estimate the position itself, or another entity can resolve it.

### 5.1. Examples

The IPSs in [24,47], rows nine and ten in Table 2, employ infrastructure modules to inject positioning purpose tones in the building’s electrical wiring at different locations. The tones (sinusoidal waves) traveling through the wiring produce EMR. The amplitude and phase of the EMR resulting from tones are expected to vary in space according to the building blueprint, the wiring itself (placement, density, and other characteristics), and the tone injection point. If the target measures the amplitudes and phases of the tones, its position could be resolved using fingerprinting. The viability of such a scheme depends on acquiring the EMR produced by the tones, and the techniques depend on the frequency of the reference tones. Section 5.5 below brings a few possibilities.

In principle, using more tones and injecting them at more points increases the reliability and diversity of the features and, consequently, the positioning accuracy. Therefore, refs. [47,78] extend the two tones (30 kHz and 447 kHz—i.e., in the 10–500 kHz) used in [24,79] to 44 tones (ranging from 447 kHz to 20 MHZ). For cells with 3–4 m sides, the two-tone PLPS achieves room-level accuracy (LC in terms of a room) and sub-room-level accuracy (position inside the room) in the ranges of 78–100% and 87–95%, respectively; and, using a 2–3 m grid for the 44-tones PLPS, the authors report accuracy gains in the range of 12–21%.

### 5.2. System Model

The first IBTB PLPS approach [24] injects specific tones in the mains to be irradiated by the cables. They are detected and sensed using a loop antenna for the fingerprint. This is the basic model for the IBTB PLPS.

**Figure 9:**
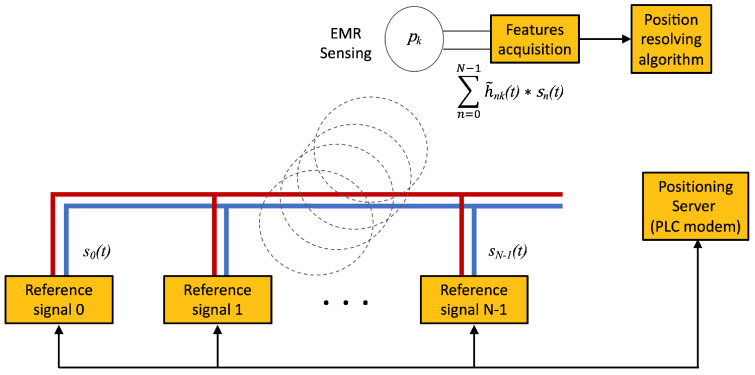
Basic scheme for Infra-based Target-based PLPS.

The infrastructure for the injection of the reference signals for IBTB PLPS is similar to the one of the IBTO PLPS. This is depicted in Figure 9. The infrastructure devices connect to the wiring to inject signals that provide the references from which the features are obtained by the target for positioning. In comparison with the IBTO, the difference resides on the collection of the features that in IBTB is accomplished by the target.

### 5.3. Positioning Features

In IBTB PLPS, the target must collect the effects of the EMR produced by the injected signal references to resolve the position. With reference to Figure 9, at the position *k*, the collected signal is

(7) $$r_{k}\left(t\right)=\sum_{n=0}^{N-1}{\tilde{h}}_{nk}\left(t\right)*s_{n}\left(t\right).$$

Note that ${\tilde{h}}_{nk}\left(t\right)$ is the hybrid wiring-wireless channel response from the signal origin *n* to the position *k* and is expected to vary with the position. Each ${\tilde{h}}_{nk}\left(t\right)$ accounts for both the wired and the wireless channels from injection point *n* to the spatial point *k* where the EMR is measured, which is the position that the system must obtain. If one assumes that one can separate the sources, which can be achieved by time or frequency multiplexing, the set of positioning features for the target at a position *k* is given by

(8) $$\begin{array}{c} f_{k}={\left\{\mathrm{features}({\tilde{h}}_{nk}\left(t\right))\right\}}_{n=0\ldots N-1}. \end{array}$$

From the collection of ${\tilde{h}}_{nk}\left(t\right)$, one obtains the position (*k*)-dependent features as the impulse response ${\tilde{h}}_{nk}\left(t\right)$ itself, the modulus of the correspondent frequency response ${\tilde{H}}_{nk}\left(f\right)$, its values at defined reference frequencies or tones *c*, where ${\tilde{H}}_{nk}\left(c\right)$, c∈C (C) is the set of tones used for the IBTB PLPS, or the tones phase differences, for example.

The diversity of features depends on using more injection points for reference signals or extracting more features from each signal. Meanwhile, in the IBTO model, one can also extend the number of sensing points to produce more features. Nevertheless, as for the IBTO PLPS, the coverage, reliability, and accuracy of IBTB PLPS depend on the number of reference carriers, where they are injected, and the wires where they flow that may make tones arrive and be sensed at different places in the building; i.e., their coverages differ. Consequently, the more tones and the greater their powers, the more extensive the combined coverage is expected to be, meaning that *k* may index a larger area.

Reliability and accuracy are expected to increase the more tones there are composing the reference signals and the more reference insertion points *n* that are employed, which is due to the better and more consistent measurements resulting from using more carriers and a larger dispersion of the entry points.

We note that different targets may present different measurements ${\tilde{h}}_{nk,\mathrm{target}}$; this cross-device effect can be accounted for introducing a target-dependent gain $G_{\mathrm{target}}$ and computing ${\tilde{h}}_{nk}^{\prime }={\tilde{h}}_{nk,\mathrm{target}}\left(t\right)/G_{\mathrm{target}}$ to obtain the features (although we exemplified only gain target differences, the approach can be extended to consider differences in frequency response as well).

The architecture of IBTB PLPS using the irradiated EMR from signals traveling in the power wiring resembles positioning efforts using the leak of RF signals from coaxial cables [80,81]. There are two correlated differences: the signal bandwidth (much higher and maybe larger) and the cable employed (coaxial). The coaxial cables are prepared to irradiate the RF signal satisfactorily, while IBTB PLPS intends to use the existing mains infrastructure.

### 5.4. IBTB PLPS Using PLC

PLC technology, Appendix A, is available for smart grids, smart cities, smart buildings, Industry 4.0, and IoT services that demand positioning services. Therefore, we analyze how PLC may support IBTB PLPS.

In this case, the signal inserted in each injection point *n* is given by Equation (4). The PLC signal has a tone-based structure that can favor feature extraction.

PLC modems connect using differential mode currents flowing through the wiring. However, Common-Mode (CM) current arises due to line imbalance [82,83,84,85] producing undesired EMR. As a result, PLC modems must comply with Electromagnetic Compatibility (EMC) regulations that limit their EMR [25,86,87,88,89]. Several works report experiments on the EMR resulting from PLC signals [51,82,83,84,85,90,91]. Nevertheless, one finds some efforts to measure the PLC EMR and characterize the PLCWC due to EMC regulations and concerns about PLC connections eavesdropping.

The EMR resulting from CM propagation of PLC signals presents intensity with varying spatial patterns [84,88]. Consequently, the EMR resulting from PLC may be used to construct an IBTB PLPS, as depicted in Figure 8. This entails acquiring features from the PLC-Wireless Channel (PLCWC), which is the channel resulting from using a PLC modem as a transmitter, while reception depends on detecting the resulting EMR [52,53]. If the target captures the PLC EMR, some subcarriers of the OFDM symbols can encode information for positioning purposes. Notwithstanding, regardless of the PLC standard and operation environment, fingerprinting methods seem to be the most reasonable approach to resolving the target position due to the difficulty in modeling the PLCWC.

### 5.5. Sensing the PLCWC

Techniques to detect and measure and extract the channel response or channel state information (CSI) from the PLCWC are necessary for designing a PLC IPS. Although the frequency band and the wire length elude the far-field assumption, we revisit some efforts that report successful techniques for the characterization of the PLCWC.

One approach to obtain the PLCWC CSI is by means of a loop antenna [47,51,52,84,86,88,91]. For example, in [47], a broadband loop antenna of 1 m in diameter is utilized for sensing 44 tones ranging from 447 kHz to 20 MHZ injected in the power line for an IBTB PLPS using fingerprinting. A 10 cm loop antenna is employed in [51] to characterize the PLCWC inside a building, and [52] uses a 30 cm loop antenna for measurements up to 40 MHz; they conclude that PLC is prone to eavesdropping.

Another approach can be seen in [53], where the authors employ omnidirectional and monopole antennas to measure the PLCWC in the 1.7 to 100 MHz band. They mention experiments inside different buildings and distances between the wiring and the antenna up to 6.0 m. They conclude that in the BB-PLC bandwidth, the PLCWC presents severe attenuation and frequency selectivity.

From the initiatives to characterize the PLCWC and obtain the CSI, as seen in rows 12 to 14 in Table 2, one sees that it is possible to sense the EMR from power line signals in the MB- and BB-PLC frequency bands. As a result, we advocate that it is possible to use the PLCWC for indoor positioning.

### 5.6. Resolving the Position

For any of the above features derived from $h_{nk}\left(t\right)$, fingerprinting (see Section 3.4) can be the means for effectively resolving the position of IBTB PLPS, since it is unfeasible to predict the EMR resulting from carriers traveling through a building cabling.

### 5.7. Hybrid PLPS Using PLC Technology

Hybrid channels may relay the PLC signals to provide the positioning features. Below, we delve into some possibilities.

#### 5.7.1. PLC–IPS Using Magnetic Beacons

**Figure 10:**
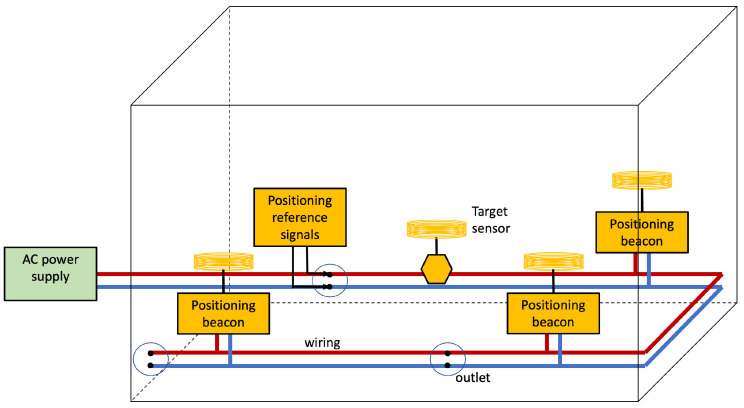
IBTB PLPS architecture using magnetic beacons. The PLPS components are highlighted in yellow. The electrical network delivers the positioning beacon to the anchor coils.

Power lines can deliver the signals for magnetic positioning. This system model is as in Figure 9, but instead of using the EMR produced by the reference signals flowing in the wiring, coils are used to irradiate positioning beacons. This scenario is depicted in Figure 10. This can be deployed by using low-frequency PLC carriers to drive the coils producing the magnetic beacons for positioning. The target also embarks coils for sensing the resulting magnetic fields. The ranges or locality identities can be obtained from the detected field.

The intensity of the voltage induced in the target coil depends on the relative position between the target and the anchor. For example, if both coils have the same specifications and orientations, the target–anchor distance follows [49]

(9) $$\begin{array}{c} d=10^{-\frac{\log\left(V_{\mathrm{target}}\right)-\log\left(\frac{\omega \mu_{0}mNr^{2}}{4}\right)}{3}}, \end{array}$$

where $V_{\mathrm{target}}$ is the voltage induced at the target coil, ω is the angular frequency of the carrier, $\mu_{0}$ is the vacuum magnetic permeability, *m* is the modulus of the magnetic moment ($\pi NI_{\mathrm{anchor}}r^{2}$), *N* is the number of turns, $I_{\mathrm{anchor}}$ is the current in the anchor coil, and *r* is its radius. Alternatively, commercial magnetic sensors can also be used to measure the magnetic field.

**Figure 11:**
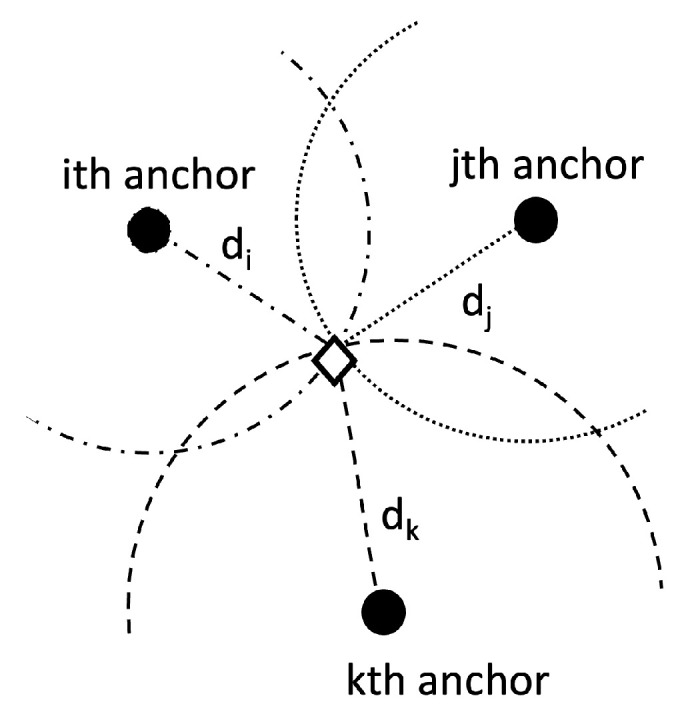
Geometrical model for range-based multilateration. To resolve the position of the target (represented by the rhomboid), one must find where the circles intercept.

From the ranges to different anchors, circular multilateration leads to the position estimate, as shown in Figure 11 [2]. The solution is given by the interception of the circles centered at the anchors.

Using this paradigm, ref. [48] employs reference signals around 189 kHz to excite the coils [49], around 24 kHz, and [50], at 125 kHz. These carriers fall within the NB-PLC frequency bands. One or multiple coils (and resonators to extend the operating range) can be used in the anchors and the target, and one or multiple carriers can be produced, simultaneously or sequentially for magnetic-field-based ranging and positioning [12,48,50,92,93,94,95,96].

#### 5.7.2. PLC–Wireless Integration

**Figure 12:**
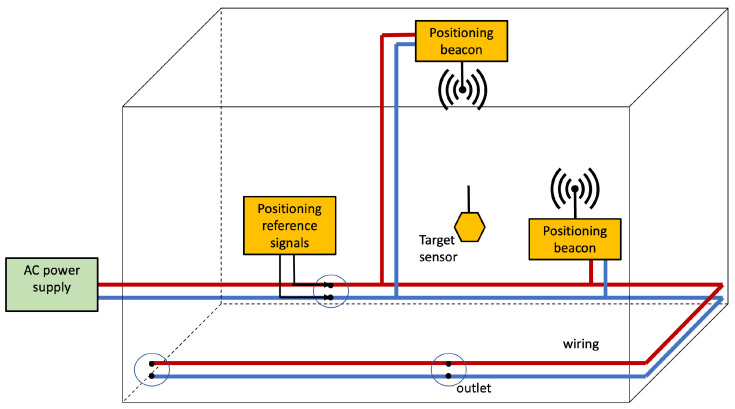
Infra-based and Target-based PLC-Wireless PLPS architecture. The PLPS components are highlighted in yellow. The electrical network delivers the positioning beacon to the anchors. Although the figure shows two positioning anchors that emit the RF beacons for positioning, more may be employed.

The Hybrid PLC–Wireless Channel (HPLCWC) is the tandem of wired and wireless links [97,98,99,100]. The PLC signal goes wireless upon the appropriate frequency shift (and the possible rearrangement of the OFDM frame). This system model is illustrated in Figure 9, but instead of using the EMR produced by the reference signals flowing in the wiring, antennas are used to irradiate positioning beacons. This leads to the IBTB PLPS depicted in Figure 12. We highlight that the HPLCWC uses the frequency band employed by wireless communications systems, whereas the PLCWC lies in the PLC frequency band. In this case, the many strategies existing for RF-signal detection and positioning in wireless networks (fingerprinting, multilateration, and multiangulation) can be used [1,2].

#### 5.7.3. PLC–VLC Integration

The VLC technology [101,102,103] turns illumination fixtures into high-capacity downlink connections. The high interference immunity to other electromagnetic sources and opaque walls favor VLC and its channel reuse indoors, respectively.

In [104], lighting fixtures emit identifications for locality classification. If the light conveys the appropriate information, the ambient light can support LC [11,15,16]. Positioning algorithms using light-based ranging, the incident angle of the light, and its intensity can also be employed [11,15,16]. Even though the identity can be assigned directly for each light bulb, it can also be set in a coordinate fashion by the PLC system. Utilizing the PLC–VLC hybrid channel to emit the positioning beacons traveling in the power lines is also possible [105].

**Figure 13:**
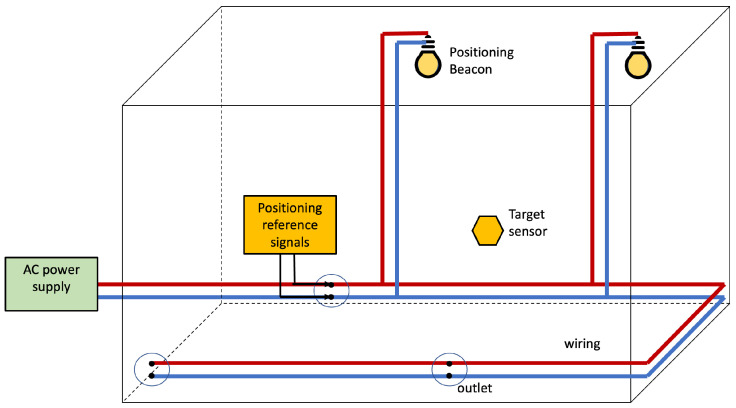
Infra-based and Target-based PLC–VLC PLPS architecture. The PLPS components are highlighted in yellow. The electrical network delivers the positioning beacon to the light anchors for transmission.

Such a hybrid PLC–VLC PLPS presents a basic model as in Figure 9, but instead of using the EMR produced by the reference signals flowing in the wiring, light sources are used to irradiate positioning beacons. The PLC–VLC PLPS is illustrated in Figure 13. Light fixtures emit the beacons, and the target employs photo-diodes or image sensors to sense the VLC channel and collect the positioning features.

### 5.8. Applications

The scenario and strategies discussed in this section encompass IBTB positioning systems that measure features from signal references injected in the electrical wiring and irradiated directly by the power line or through hybrid approaches.

Some experiments listed in Table 2 indicate the possibility of measuring the PLCWC and extracting the CSI for MB- and BB-PLC systems, which, for example, can be used for fingerprinting-based positioning.

Using ranging from magnetic beacons for IBTB PLPS has the advantage that magnetic coupling at low frequencies is reasonably robust to people and changes in room configuration.

The PLC-wireless and PLC–VLC hybrid approaches may benefit from frame structures that simultaneously fit PLC and WiFi or PLC and VLC. To this end, frame structures and transceivers that can jointly be used for positioning and communications in BB-PLC, WiFi (wireless), and VLC systems are necessary to back the utilization of the same processing chain for the different physical channels, resulting in a simpler target design and lowering the cost. Meanwhile, the construction of PLC-IPS using Magnetic Fields in Section 5.7.1 could perfectly use NB-PLC carriers.

The many applications of IPS encountered today may rest on the different IBTB PLPS architectures above discussed.

## 6. Some Comments on the Deployment of PLPS and Application Scenarios

Section 2 discussed the characteristics and properties of the three PLPS architectures. We note that although all PLPS architectures aim at a position estimate, their architectures and possible uses are rather different. Thus, in the previous sections, we have pointed out the different positioning features that the different PLPS categories may rely on, how to collect them, and general use-case scenarios and applications for the three PLPS categories.

While the technology of ILTB PLPS relies solely on the power line infrastructure and the energy it delivers, the IBTO and IBTB use the power lines to irradiate or, in some cases, simply conduct the positioning signals for the irradiation by a magnetic, RF, or light transducer. Since the electrical wiring is not in principle intended for data transfer, the bandwidth for the positioning signals presents severe restrictions. Both the IBTO and IBTB PLPS must comply with Electromagnetic Compatibility (EMC) regulations [25,86,87,88,89] that limit the electromagnetic emissions, as any other equipment. PLC modems are designed to effectively use the wiring for communications and to comply with EMC regulations and thus appear as an adequate means for providing the PLPS infrastructure for IBTO and IBTB PLPS. ILTB PLPS do not introduce any positioning reference into the power line; they are autonomous systems that opportunistically employ any aspect of the existing power system for positioning purposes. Section 3 discussed ILTB systems architectures, possible features, and strategies that may leverage autonomous robots and other self-positioning applications. In an ILTB PLPS, the power lines passively provide the positioning features. Being infrastructure-free, such a system can be deployed in many scenarios, although the positioning accuracy may largely vary depending on the features employed for positioning, and, in many cases, switching among different sensors and features may be necessary for accurate positioning.

The IBTO PLPS turns about the ILTB, since the target is passive. An IBTO PLPS rests on how the target affects the signals traveling through the wire lines. Section 4 discussed possible IBTO systems architectures, features, and strategies that may, for example, leverage the obtrusive positioning of people. However, small regions and the small bandwidth of the power lines may hamper to obtain features of sufficient diversity for accurate positioning, as we describe below in Section 6.1; again, PLC signals may be a reasonable approach to increase feature diversity for effective IBTO PLPS implementations.

Since an IBTB PLPS requires equipment on both sides, at the infrastructure to produce the positioning references and at the target for their acquisition, one may say either that an IBTB PLPS lies between ILTB and IBTO or encompasses both. Section 5 discussed IBTB systems architectures, possible features, and strategies; the main conclusion is that the direct use of PLC signals traveling through the power lines may be very difficult if not unfeasible due to the long wavelength and faint intensity of the EMR produced by the PLC. Consequently, a probable scenario for building IBTB PLPS is using the BB-PLC signals to deliver the references for emission using RF or light in a hybrid fashion. Since magnetic coupling positioning can be precise and accurate and is immune to the effects of the human body, another possibility is using NB-PLC carriers through the wiring to excite coils for magnetic positioning in large storage buildings and alike.

Table 2 brings the reaches for feature collection for the different PLPS initiatives. From the data in the literature and the working principles of the different PLPS architectures, it is possible to speculate on their accuracy as well. ILTB systems depend on the sensors employed and can switch among them; when images are employed, centimeter-level accuracy can be expected; meanwhile, at very close distances from a power outlet, millimetric accuracy can be possible. The development and improvement of PLPS IBTB systems using signals traveling through the power lines may lead to an accuracy of meters or even tenths of centimeters. For hybrid IBTB PLPS, the accuracy depends on the second hop that irradiates the beacons. For magnetic-based positioning, centimeter-level accuracy is expected. Using OFDM symbols for RF-based IBTB PLPS, the accuracy can be as high as for WiFi-based positioning systems. If the light is employed instead, accuracy improvements can be obtained. Regarding IBTO PLPS, distinguishing the target position is hardened due to the nature of the collected features. Consequently, the accuracy may be limited to meters, making IBTO PLPS more suitable for location classification than providing a position estimate.

### 6.1. Unobtrusive PLPS Using PLC

PLC modems are already in the market. The transmitter injects the PLC signal into the wiring, and the receiver detects and demodulates it. Consequently, PLC systems may seamlessly provide the infrastructure for the IBTO PLPS. Since the BB-PLC signals lie within a bandwidth where the human–body–conductor coupling may be important, changes in the channel responses for the OFDM subcarriers of BB-PLC can be used for presence detection and possibly LC and positioning. Since PLC and smart metering using PLC are increasingly deployed [106,107,108,109,110], the technology may already be available at several premises.

### 6.2. Positioning in Ships

In [111,112], the authors claim that accurate and pervasive positioning in cruiser ships improves the response to incidents. For example, it can help evacuation routing to reduce hazards during incidents [113]. Other relevant tasks are detecting and counting the workforce in ships’ bridges for safety watchkeeping [114]. Recently, ref. [115] highlights that positioning systems on shipboards could keep track of passengers to identify areas of high transmission risk of infection in cruise ships. Designing PLPS for shipboards may be favored, as PLC systems have been utilized in ships for different applications, most remarkably for the communication among the different automation systems embarked [116,117,118]. Since power is vertically distributed in cruise ships with a reduced number of rooms per segment [118], an unobtrusive PLPS may help to determine the busiest areas on ships. Nonetheless, in-ships, the power distribution may employ armored or unarmored cables [119], affecting the channel response and the resulting EMR.

### 6.3. Underground Mines

Safety concerns apply to underground mines, and positioning can help to reduce hazards. However, the underground mine is a hard environment for positioning systems [22,120]. Variants of existing IPS technologies have been tried in this scenario, but such an environment hampers using acoustic and RF-based positioning systems. Similar arguments indicate that the same obstacles exist in risky construction sites, which are a defying situation for positioning systems [19].

In mines, power must be delivered for different tasks. The deployed infrastructure can be used to back PLPS. Both IBTO and IBTB PLPS can be used in this scenario. For the IBTO PLPS, the concepts already discussed apply as well in underground mines. For deploying IBTB PLPS in underground mines, both magnetic beacons and hybrid PLC–VLC are of interest to provide ranging-based and locality classification.

### 6.4. Industry 4.0

Industry 4.0 is expected to result from massive sensor deployment, high device connectivity, automation, and real-time data acquisition to evolve from computerized manufacturing to cyber–physical systems (physical objects with embedded software and computing power) collaboration. The manufactured products are expected to become smart [121] with self-management capabilities. The Industry 4.0 paradigm aims at improving the industrial process to [122]: (i) fulfill individual customer requirements profitably, (ii) provide flexibility to rapidly respond to disruptions and failures, (iii) achieve transparent decision making of the manufacturing processes, and (iv) automate processes subject to resource and energy efficiency constraints. Many objectives require positioning services at a large scale and low cost for operators, tools, materials, products, and other objects and parts of the production chain to [18,123,124,125,126,127,128,129]: (i) reduce unnecessary displacements, (ii) optimize the layout and the supply chain, (iii) reduce exposure to material and material handling, (iv) support real-time manufacturing, (v) track the tools and their use to optimize their placement and storage, and (vi) reduce stock and waste; to list a few. The required positioning accuracy for Industry 4.0 services may range from simple presence detection and locality identification up to sub-cm accuracy.

Different cabling technologies are used in factories following many standardized procedures. They may be employed to support PLPS in such premises. The different electrical system components provide visual and heat-pattern clues that can be used for ILTB autonomous positioning. In the manufacturing lines that employ a small working force, one may employ IBTO PLPS to locate the workers. Meanwhile, the environment may be defiant: as more machines are used, their powers increase, and their operating cycles vary over time, among other issues. In such scenarios, the hybrid PLC-based IBTB PLPS may help to provide positioning services using the cables as the backbone for the positioning system.

### 6.5. Hybrid and Fusion PLPS

Power lines can partake in hybrid and fused positioning systems [130,131,132]. For example, one may employ a compass, magnetometers, ambient light, pressure, and data from different RF anchors (mobile networks, WiFi, Bluetooth, ZigBee, among others) for a hybrid fingerprint. In addition, light and magnetic ranging could be mixed for multilateration. Alternatively, fingerprinting position estimates can be combined with dead reckoning. These examples illustrate that different features can be combined for hybrid computation of the position estimates or that fused position estimates can also be approached; increasing the diversity of features and positioning strategies extends the availability, reliability, and accuracy of the positioning system.

## 7. Conclusions

This prospective survey mapped how power lines can support positioning services and some of the potential applications for power line positioning systems (PLPS). We devised three main system architectures or classes depending on whether the system uses specific infrastructure to produce reference positioning signals and whether the target makes measurements to collect the features employed to estimate its position. Infra-less Target-based PLPS architecture can be employed for self-positioning by different devices; Infra-based Target-obtuse PLPS architecture can be employed for obtrusive positioning of persons; and, Infra-based Target-based PLPS architecture can be employed for general positioning applications.

Since Power Line Communication (PLC) systems employ the mains infrastructure for communications, this work has also indicated approaches for using PLC to implement Infra-based PLPS. We mapped the PLC spectral bands appropriate for the IBTO and IBTB PLPS architectures that may foster deploying PLC-based indoor positioning systems (PLC-IPS). The development of PLPS is still ongoing and requires more experiments, proposals, and developments. Strategies, devices, sensors, and methods to employ PLC signals emitted directly by the power lines or using a hybrid medium for effective positioning need to be further investigated. We hope this contribution helps PLPS research and development by providing relevant directions for these tasks.

## Figures and Tables

**Table 1:** Categories, characteristics, and broad application scenarios of positioning systems using the mains. The category indicates if the PLPS employs specific infrastructure to produce and inject reference signals or not (based × less) and if the target is involved in the positioning process or not (based × obtuse). The characteristics specify if and where the reference signals are produced, if they may come from PLC, which system part collects the positioning features, and where is the position resolved. The application scenarios are self-, unobtrusive, and transparent positioning.

Category	Characteristics					Application Scenarios		
	Reference Signals		Sensor Location	Position Computation		Self-Positioning	Unobtrusive	Transparent
	Exist	PLC-born		Where	Cooperation			
**Infra-less Target-based** (ILTB)	No	No	Target	Target	No	Yes	Not Applicable	Not Applicable
**Infra-based Target-obtuse** (IBTO)	Yes	Possible	Infra	Infra	No	No	Yes	Possible
**Infra-based Target-based** (IBTB)	Yes	Possible	Target	Infra or Target	Possible	No	No	Yes

**Table 2:** List of PLPS and experiments with results indicating possibilities for PLC-IPS design.

Purpose	Category	Feature Principle	Feature Source	Instrument/Sensor	Reach	Utilization	PLC Band	Ref.
Robot plugging-in (Self-positioning)	ILTB	EMR	AC carrier, LV	E-filed detector	cm	Outlet detection from the E-field	None	[42]
Robot plugging-in (Self-positioning)	ILTB	Image processing	Outlet Image	Camera	mm	Outlet alignment using image processing	Not applicable	[42]
Robot plugging-in (Self-positioning)	ILTB	EMR	AC carrier, LV	Copper foil	cm	Outlet detection detection from the E-field	None	[32]
Robot plugging-in (Self-positioning)	ILTB	EMR	AC carrier, LV	Ground prong	mm	Outlet alignment detection using the E-field	None	[32]
UAV perching (Self-positioning)	ILTB	EMR	AC carrier, MV	Magnetometer	cm and m	Relative distance	None	[43,44]
SLaM (Self-positioning)	ILTB	EMR	AC carrier, LV	Tank circuit and microphone	m	Fingerprint positioning	None	[45]
Locality classification (Unobtrusive)	IBTO	Body-wiring coupling	70 MHz carrier	Voltage peak detector	m	Fingerprint positioning	Greater than BB-PLC	[35,46]
IPS	IBTB	EMR	30 and 447 kHz carriers	Tank circuit	m	Fingerprint positioning	NB-PLC	[24]
IPS	IBTB	EMR	44 carriers (0.447–20 MHz)	Loop antenna of 1 m	m	Fingerprint positioning	MB-PLC BB-PLC	[47]
IPS	IBTB	Induced voltage	24, 125, 189 kHz	Coils and resonators	cm and m	Ranging and multilateration	NB-PLC	[48,49,50]
Eavesdropping	None (IBTB)	EMR	1–40 MHz band	Loop antennas of 10 and 30 cm	m	Channel response (CSI)	MB-PLC BB-PLC	[51,52]
Capacity evaluation	None (IBTB)	EMR	1.7–100 MHz band	Monopole antenna	m	Channel response (CSI)	MB/BB-PLC	[53]
Capacity evaluation	None (IBTB)	EMR	10–30 MHz band	Coupled inductors	cm	Channel response (CSI)	BB-PLC	[54]

**Figure A1:**
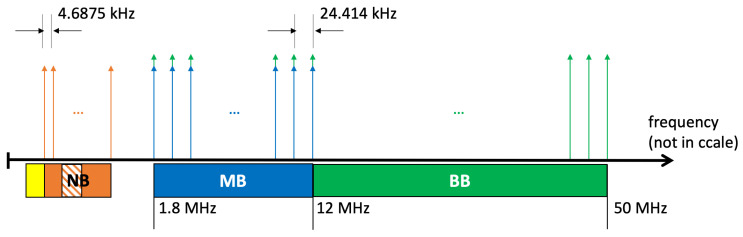
Frequency bands for the IEEE PLC standards and OFDM subcarriers separation. Notations NB, MB, and BB refer to narrow band, medium band, and broad band, respectively. Notice that the BB frequency range also includes the MB band.

## Abbreviations

The following abbreviations are used in this manuscript:

AC	Alternate Current
ARIB	Association of Radio Industries and Business
BB	Broad Band
BPSK	Binary Phase Shift Keying
CENELEC	European Committee for Electrotechnical Standardization
CM	Common Mode
CSI	Channel State Information
DC	Direct Current
EMF	Electromagnetic Field
EMR	Electromagnetic Radiation
FCC	Federal Communications Commission
FFT	Fast Fourier Transform
GPS	Global Positioning Systems
HPCLWC	Hybrid PLC-Wireless Channel
HV	High Voltage
IBTB	Infra-Based Target-Based
IBTO	Infra-Based Target-Obtuse
ILTB	Infra-Less Target-Based
IEEE	Institute of Electrical and Electronic Engineers
IFFT	Inverse Fast Fourier Transform
IPS	Indoor Positioning System
LAN	Local Area Networks
LC	Locality Classification
LV	Low Voltage
OFDM	Orthogonal Frequency Division Multiplexing
MB	Medium Band
MV	Medium Voltage
NB	Narrow Band
PAM	Pulse Amplitude Modulation
PLC	Power Line Communication
PLC-IPS	PLC-Based IPS
PLCWC	PLC Wireless Channel
PLPS	Power Line Positioning System
QAM	Quadrature Amplitude Modulation
QPSK	Quadrature Phase Shift Keying
RF	Radio Frequency
SLaM	Simultaneous Localization and Mapping
UAV	Unmanned Aerial Vehicle
UNB	Ultra Narrow Band
VLC	Visible Light Communication
VLP	Visible Light Positioning

## Appendix A. PLC Categories

The power lines present different characteristics [25,133,134,135,136] in the transmission (High Voltage—HV), distribution (Medium Voltage—MV), and delivery (Low Voltage—LV) sections. Consequently, the channel varies with the voltage section, and its response can be frequency selective and time-varying. It depends on the number of connected lines and devices, the line segment types and their lengths, and the type of devices. PLC standards employ multi-carrier approaches for narrow-band, medium-band, and broad-band communications [25,137,138,139,140,141,142].

There are four PLC categories depending on their bandwidths (and the envisioned application scenario): Ultra Narrow Band (UNB), Narrow Band (NB), Medium Band (MB), and Broad Band (BB). The frequency bands used by the PLC IEEE standards are illustrated in Figure A1. Using OFDM in the physical layer (PHY), the available bandwidth is divided into several subchannels. Figure A1 also presents the spacing between OFDM subcarriers for the IEEE PLC standards. Meanwhile, time-division multiple access is most frequently used for channel access and medium sharing by multiple hosts. UNB-PLC systems use a narrower bandwidth than the NB-PLC, being, in general, proprietary. Following, we briefly present the IEEE PLC standards, but other forums [141,142] propose similar PLC profiles.

**Table A1:** IEEE BB-PLC Standard 1901 FFT OFDM and wavelet OFDM PHYs major specifications.

	FFT OFDM PHY	Wavelet OFDM PHY
**Forward Error Correction**	Turbo-convolutional coding	Reed–Solomon coding
		Convolutional coding
		Concatenated coding
		Low-Density Parity-Check convolutional coding (optional)
**Primary Modulation**	BPSK, QPSK, 8-QAM to	2-PAM to 16-PAM, 32-PAM (optional)
	1024-QAM, 4096-QAM (optional)	
**Subchannels (*C*)**	4096 (917 active)	512, 1024, 2048
**Prototype Filter Length (2*qC*)**	–	*q* = 2, 3, 4
**Frequency Band**	1.8–50 MHz	1.8 MHz–28 MHz, 1.8 MHz–50 MHz (optional)
**Symbol Length (μs)**	40.96	8.192, 16.384 (optional), 32.768 (optional)

### Appendix A.1. Broadband-PLC

The BB-PLC IEEE Std 1901 “IEEE Standard for Broadband over Power Line Networks: Medium Access Control and Physical Layer Specifications” [138] aims at high-throughput communications local area networks (LAN) to compete against other wired and wireless LAN technologies. The electric power lines acting as communication media may carry Alternating Current (AC) at a low and medium voltage, Direct Current (DC), or be non-energized both outside and inside commercial and residential buildings and factory premises.

The IEEE 1901 BB-PLC specifies two physical-layer procedures, as listed in Table A1. The standard relies on OFDM, using up to 917 carriers, ranging from 1.80 to 50.00 MHz. The OFDM symbol is obtained using a 100 MHz sampling clock, a 4096-point IFFT, and cyclic-prefix addition. In the so-called full mode, the carrier spacing is approximately 24.414 kHz. The symbol duration is 40.96 μs. Each carrier is the smallest unit of transmission and transports coherent symbols: Binary Phase-Shift Keying (BPSK), Quadrature Phase-Shift Keying (QPSK), 8-Quadrature-Amplitude Modulation (QAM), 16-QAM, 64-QAM, 256-QAM, or 1024-QAM, and optionally 4096-QAM modulation. Alternatively, the standard deploys wavelet OFDM PHY [143,144]. In the wavelet OFDM PHY, each carrier may convey one bit using two Pulse Amplitude Modulation (2PAM), two bits using 4PAM, three bits using 8PAM, four bits using 16PAM, or five bits using 32PAM.

### Appendix A.2. Medium-Band-PLC

The MB-PLC IEEE Std 1901.1 “IEEE Standard for Medium Frequency (less than 12 MHz) Power Line Communications for Smart Grid Applications” [139] aims at the power line support of the necessary capacity for smart-grid applications using the same channels as the BB-PLC IEEE Std 1901.

The IEEE 1901.1 MB-PLC specifies OFDM symbols using the spectrum between 1.80 and 12 MHz. The time symbol is based on a 25 MHz sampling clock and a 1024-point IFFT. A cyclic prefix is inserted to create the symbol. The carrier spacing equals the BB-PLC spacing, which is approximately 24.414 kHz. The symbol duration is 5.12 or 10.24 μs, respectively, an eighth and a fourth of the duration of the BB-PLC OFDM symbol.

### Appendix A.3. Narrowband-PLC

The IEEE Std 1901.2 “IEEE Standard for Low-Frequency (less than 500 kHz) Narrowband Power Line Communications for Smart Grid Applications” [140] aims at Smart Grid and low-bandwidth connections (at most 500 kbps): grid to the utility meter, grid automation, electric vehicle to charging station, lighting, solar-panel, and home-area networking. The IEEE 1901.2 NB-PLC supports connections over DC and AC power lines over LV (less than 1000 V) and MV (1000 V to 72 kV) (MV) power lines and through transformers. A node in the MV section should communicate with nodes in the LV section even after stepping down from MV to LV and vice versa for urban and long-distance rural connections.

The IEEE 1901.2 NB-PLC specifies narrow-band communications in the frequency band up to 500 kHz. It assures coexistence with broad-band power line devices by minimizing out-of-band emissions in frequencies greater than 500 kHz. The NB-PLC employs frequencies between 10 and 490 kHz, accommodating the requirements of different regulations boards: the Federal Communications Commission (FCC), the European Committee for Electrotechnical Standardization (CENELEC), and the Association of Radio Industries and Businesses (ARIB). Thus, the NB-PLC operates in the bands from 35.9375 to 90.625 kHz, CENELEC A, and from 98.4375 to 121.875 kHz, CENELEC B, from 37.5 to 117.1875 kHz, FCC-low, and from 154.6875 to 487.5 kHz, FCC-above-CENELEC, from 37.5 to 117.1875 kHz, ARIB 1, and from 154.6875 to 403.125 kHz, ARIB 2.

The minimum sampling frequency at the transmitter is 1.2 MHz. The maximum number of carriers within 10 kHz and 490 kHz is 128 (36 in the CENELEC-A band), resulting in a minimum IFFT size of *N* = 256. For example, in the FCC and ARIB bands, this results in a frequency spacing between the OFDM carriers equal to 4.6875 kHz. Out-of-band tones are masked. The cyclic prefix contains the final 30 samples. Each carrier can carry Phase Shift Keying (PSK) or QAM symbols (support is mandatory for differential BPSK, differential QPSK, and differential 8PSK, and it is optional for BPSK, QPSK, 8PSK, and 16QAM).

## References

[1] Zekavat R., Buehrer R.M. (2011). Handbook of Position Location: Theory, Practice and Advances.

[2] Campos R.S., Lovisolo L. (2015). RF Positioning: Fundamentals, Applications, and Tools.

[3] Zekavat S.R., Buehrer R.M., Durgin G.D., Lovisolo L., Wang Z., Goh S.T., Ghasemi A. (2021). An Overview on Position Location: Past, Present, Future. Int. J. Wirel. Inf. Netw..

[4] Pannuto P., Kempke B., Chuo L.X., Blaauw D., Dutta P. (2018). Harmonium: Ultra wideband pulse generation with bandstitched recovery for fast, accurate, and robust indoor localization. ACM Trans. Sens. Netw. (TOSN).

[5] Becker C., Dürr F. (2005). On location models for ubiquitous computing. Pers. Ubiquitous Comput..

[6] Mautz R. (2012). Indoor Positioning Technologies.

[7] Harle R. (2013). A survey of indoor inertial positioning systems for pedestrians. IEEE Commun. Surv. Tutor..

[8] Ijaz F., Yang H.K., Ahmad A.W., Lee C. Indoor positioning: A review of indoor ultrasonic positioning systems. Proceedings of the IEEE 2013 15th International Conference on Advanced Communications Technology (ICACT).

[9] Davidson P., Piché R. (2016). A survey of selected indoor positioning methods for smartphones. IEEE Commun. Surv. Tutor..

[10] Brena R.F., García-Vázquez J.P., Galván-Tejada C.E., Muñoz-Rodríguez D., Vargas-Rosales C., Fangmeyer J. (2017). Evolution of indoor positioning technologies: A survey. J. Sens..

[11] Luo J., Fan L., Li H. (2017). Indoor positioning systems based on visible light communication: State of the art. IEEE Commun. Surv. Tutor..

[12] Pasku V., De Angelis A., De Angelis G., Arumugam D.D., Dionigi M., Carbone P., Moschitta A., Ricketts D.S. (2017). Magnetic field-based positioning systems. IEEE Commun. Surv. Tutor..

[13] Tariq Z.B., Cheema D.M., Kamran M.Z., Naqvi I.H. (2017). Non-GPS positioning systems: A survey. ACM Comput. Surv. (CSUR).

[14] Ferreira A.F.G.G., Fernandes D.M.A., Catarino A.P., Monteiro J.L. (2017). Localization and positioning systems for emergency responders: A survey. IEEE Commun. Surv. Tutor..

[15] Zhuang Y., Hua L., Qi L., Yang J., Cao P., Cao Y., Wu Y., Thompson J., Haas H. (2018). A survey of positioning systems using visible LED lights. IEEE Commun. Surv. Tutor..

[16] Lovisolo L., Tcheou M.P., Ávila F.R. (2018). Visible Light-Based Communication and Localization. Handbook of Position Location: Theory, Practice, and Advances.

[17] Zafari F., Gkelias A., Leung K.K. (2019). A survey of indoor localization systems and technologies. IEEE Commun. Surv. Tutor..

[18] Lam E.W., Little T.D. Visible light positioning for location-based services in Industry 4.0. Proceedings of the IEEE 2019 16th International Symposium on Wireless Communication Systems (ISWCS).

[19] Li C.T., Cheng J.C., Chen K. (2020). Top 10 technologies for indoor positioning on construction sites. Autom. Constr..

[20] Rafsanjani H.N., Ghahramani A. (2020). Towards utilizing internet of things (IoT) devices for understanding individual occupants’ energy usage of personal and shared appliances in office buildings. J. Build. Eng..

[21] Rocamora J.M., Ho I.W.H., Mak W.M., Lau A.P.T. (2020). Survey of CSI fingerprinting-based indoor positioning and mobility tracking systems. IET Signal Process..

[22] Seguel F., Palacios-Játiva P., Azurdia-Meza C.A., Krommenacker N., Charpentier P., Soto I. (2021). Underground mine positioning: A review. IEEE Sens. J..

[23] Kim Geok T., Zar Aung K., Sandar Aung M., Thu Soe M., Abdaziz A., Pao Liew C., Hossain F., Tso C.P., Yong W.H. (2021). Review of indoor positioning: Radio wave technology. Appl. Sci..

[24] Patel S.N., Truong K.N., Abowd G.D. (2006). Powerline positioning: A practical sub-room-level indoor location system for domestic use. Proceedings of the International Conference on Ubiquitous Computing.

[25] Lampe L., Tonello A.M., Swart T.G. (2016). Power Line Communications: Principles, Standards and Applications From Multimedia to Smart Grid.

[26] Campos R.S., Lovisolo L. (2011). RF fingerprinting location techniques. Handbook of Position Location: Theory, Practice, and Advances.

[27] Al-Ammar M.A., Alhadhrami S., Al-Salman A., Alarifi A., Al-Khalifa H.S., Alnafessah A., Alsaleh M. Comparative survey of indoor positioning technologies, techniques, and algorithms. Proceedings of the IEEE 2014 International Conference on Cyberworlds.

[28] Mudriievskyi S. (2014). Power line communications: State of the art in research, development and application. AEU-Int. J. Electron. Commun..

[29] Cano C., Pittolo A., Malone D., Lampe L., Tonello A.M., Dabak A.G. (2016). State of the art in power line communications: From the applications to the medium. IEEE J. Sel. Areas Commun..

[30] López G., Matanza J., De La Vega D., Castro M., Arrinda A., Moreno J.I., Sendín A. (2019). The role of power line communications in the smart grid revisited: Applications, challenges, and research initiatives. IEEE Access.

[31] Tsuzuki S., Takeichi N., Hamada M., Yamada Y. A Proposal of Synchronization Beacon Systems over Power-line for Indoor Fine-Grained Localization. Proceedings of the 2006 IEEE International Symposium on Power Line Communications and its Applications.

[32] Mayton B., LeGrand L., Smith J.R. Robot, feed thyself: Plugging in to unmodified electrical outlets by sensing emitted AC electric fields. Proceedings of the 2010 IEEE International Conference on Robotics and Automation.

[33] Society of Automotive Engineers International (2019). Wireless Power Transfer for Light-Duty Plug-In/Electric Vehicles and Alignment Methodology.

[34] Seong J.Y., Lee S.S. (2020). A Study on Precise Positioning for an Electric Vehicle Wireless Power Transfer System Using a Ferrite Antenna. Electronics.

[35] Zhou T., Zhang Y., Chen X., Zhang P., Zhang L. E-loc: Indoor localization through building electric wiring. Proceedings of the 16th ACM/IEEE International Conference on Information Processing in Sensor Networks.

[36] Kiedrowski P. (2015). Toward more efficient and more secure last mile smart metering and smart lighting communication systems with the use of PLC/RF hybrid technology. Int. J. Distrib. Sens. Netw..

[37] de Mello Brandao Abdo Dib L., Fernandes V., de Filomeno M.L., Ribeiro M.V. (2017). Hybrid PLC/wireless communication for smart grids and internet of things applications. IEEE Internet Things J..

[38] Ding W., Yang F., Yang H., Wang J., Wang X., Zhang X., Song J. (2015). A hybrid power line and visible light communication system for indoor hospital applications. Comput. Ind..

[39] Gheth W., Rabie K.M., Adebisi B., Ijaz M., Harris G. Performance analysis of integrated power-line/visible-light communication systems with AF relaying. Proceedings of the 2018 IEEE Global Communications Conference (GLOBECOM).

[40] Jani M., Garg P., Gupta A. (2019). Performance analysis of a co-operative PLC/VLC system with multiple access points for indoor broadcasting. AEU-Int. J. Electron. Commun..

[41] Ndjiongue A.R., Shongwe T., Ferreira H.C., Ngatched T.N., Vinck A.H. Cascaded PLC-VLC channel using OFDM and CSK techniques. Proceedings of the 2015 IEEE Global Communications Conference (GLOBECOM).

[42] Bagepalli A., Zamora M., Sánchez D. (2005). Autonomous Self-Guiding Wall Charging Robot. https://www.slideserve.com/libitha/autonomous-self-guiding-wall-charging-robot.

[43] Moore J., Tedrake R. Powerline perching with a fixed-wing UAV. Proceedings of the AIAA Infotech@ Aerospace Conference and AIAA Unmanned... Unlimited Conference.

[44] Moore J., Tedrake R. Magnetic localization for perching UAVs on powerlines. Proceedings of the 2011 IEEE/RSJ International Conference on Intelligent Robots and Systems.

[45] Lu C.X., Li Y., Zhao P., Chen C., Xie L., Wen H., Tan R., Trigoni N. Simultaneous localization and mapping with power network electromagnetic field. Proceedings of the 24th Annual International Conference on Mobile Computing and Networking.

[46] Zhou T., Zhang Y., Chen X., Mosalam K.M., Noh H.Y., Zhang P., Zhang L. P-Loc: A device-free indoor localization system utilizing building power-line network. Proceedings of the 2019 ACM International Joint Conference on Pervasive and Ubiquitous Computing and Proceedings of the 2019 ACM International Symposium on Wearable Computers.

[47] Stuntebeck E.P., Patel S.N., Robertson T., Reynolds M.S., Abowd G.D. Wideband powerline positioning for indoor localization. Proceedings of the 10th International Conference on Ubiquitous Computing.

[48] De Angelis G., Pasku V., De Angelis A., Dionigi M., Mongiardo M., Moschitta A., Carbone P. (2015). An indoor AC magnetic positioning system. IEEE Trans. Instrum. Meas..

[49] Pasku V., De Angelis A., Dionigi M., De Angelis G., Moschitta A., Carbone P. (2015). A positioning system based on low-frequency magnetic fields. IEEE Trans. Ind. Electron..

[50] Hehn M., Sippel E., Carlowitz C., Vossiek M. (2019). High-accuracy localization and calibration for 5-DoF indoor magnetic positioning systems. IEEE Trans. Instrum. Meas..

[51] Degauque P., Laly P., Degardin V., Lienard M., Diquelou L. Compromising electromagnetic field radiated by in-house PLC lines. Proceedings of the 2010 IEEE Global Telecommunications Conference GLOBECOM 2010.

[52] Degardin V., Laly P., Lienard M., Degauque P. (2011). Compromising radiated emission from a power line communication cable. J. Commun. Softw. Syst..

[53] Oliveira T., Andrade F., Picorone A., Latchman H., Netto S.L., Ribeiro M.V. (2016). Characterization of hybrid communication channel in indoor scenario. J. Commun. Inf. Syst. (JCIS).

[54] Barmada S., Tucci M., Raugi M., Dionigi M., Mezzanotte P. Experimental validation of a hybrid wireless power transfer-power line communication system. Proceedings of the 2016 International Symposium on Power Line Communications and its Applications (ISPLC).

[55] Ralchenko M., Roper M. VLF magnetic positioning in multistory parking garages. Proceedings of the 2018 International Conference on Indoor Positioning and Indoor Navigation (IPIN).

[56] Haverinen J., Kemppainen A. (2009). Global indoor self-localization based on the ambient magnetic field. Robot. Auton. Syst..

[57] Gozick B., Subbu K.P., Dantu R., Maeshiro T. (2011). Magnetic maps for indoor navigation. IEEE Trans. Instrum. Meas..

[58] Subbu K.P., Gozick B., Dantu R. (2013). LocateMe: Magnetic-fields-based indoor localization using smartphones. ACM Trans. Intell. Syst. Technol. (TIST).

[59] Rodríguez D.P.N., Apolinário J.A., Biscainho L.W.P. (2010). Audio authenticity: Detecting ENF discontinuity with high precision phase analysis. IEEE Trans. Inf. Forensics Secur..

[60] Martens S.M., Mischi M., Oei S.G., Bergmans J.W. (2006). An improved adaptive power line interference canceller for electrocardiography. IEEE Trans. Biomed. Eng..

[61] Li Y., Tan R., Yau D.K. Natural timestamping using powerline electromagnetic radiation. Proceedings of the 2017 16th ACM/IEEE International Conference on Information Processing in Sensor Networks (IPSN).

[62] Li Y., Tan R., Yau D.K. (2018). Natural timestamps in powerline electromagnetic radiation. ACM Trans. Sens. Netw. (TOSN).

[63] Ripka P. (2021). Magnetic Sensors and Magnetometers.

[64] Filippopoulos G., Tsanakas D. (2005). Analytical calculation of the magnetic field produced by electric power lines. IEEE Trans. Power Deliv..

[65] Kosba A.E., Saeed A., Youssef M. RASID: A robust WLAN device-free passive motion detection system. Proceedings of the 2012 IEEE International Conference on Pervasive Computing and Communications.

[66] Deak G., Curran K., Condell J. (2012). A survey of active and passive indoor localisation systems. Comput. Commun..

[67] das Chagas A.O.S., Lovisolo L., Tcheou M.P. Detecçao de Risco de Queda no Ambiente Hospitalar a partir da Informaçao do Estado do Canal Sem-Fio. Proceedings of the Brazilian Congress on Computational Intelligence (CBIC).

[68] Seifeldin M., Saeed A., Kosba A.E., El-Keyi A., Youssef M. (2012). Nuzzer: A large-scale device-free passive localization system for wireless environments. IEEE Trans. Mob. Comput..

[69] Jin Z., Bu Y., Liu J., Wang X., An N. Development of Indoor Localization System for Elderly Care Based on Device-Free Passive Method. Proceedings of the 2015 Sixth International Conference on Intelligent Systems Design and Engineering Applications (ISDEA).

[70] Saeed A., Kosba A.E., Youssef M. (2013). Ichnaea: A low-overhead robust WLAN device-free passive localization system. IEEE J. Sel. Top. Signal Process..

[71] Chow K.H., He S., Tan J., Chan S.H.G. (2018). Efficient locality classification for indoor fingerprint-based systems. IEEE Trans. Mob. Comput..

[72] Xiao J., Wu K., Yi Y., Wang L., Ni L.M. Pilot: Passive device-free indoor localization using channel state information. Proceedings of the 2013 IEEE 33rd International Conference on Distributed Computing Systems.

[73] Dias P.V.G., Costa E.D.M., Tcheou M.P., Lovisolo L. Fall detection monitoring system with position detection for elderly at indoor environments under supervision. Proceedings of the 2016 8th IEEE Latin-American Conference on Communications (LATINCOM).

[74] Djaja-Josko V. Presence and Fall Detection in Confined Indoor Environments Using Multiple UWB Transceivers. Proceedings of the 2018 26th Telecommunications Forum (TELFOR).

[75] Rashid K.M., Louis J., Fiawoyife K.K. (2019). Wireless electric appliance control for smart buildings using indoor location tracking and BIM-based virtual environments. Autom. Constr..

[76] Aipperspach R., Woodruff A., Anderson K., Hooker B. (2005). Maps of Our Lives: Sensing People and Objects Together in the Home.

[77] Zhan Y., Haddadi H. (2021). MoSen: Activity Modelling in Multiple-Occupancy Smart Homes. arXiv.

[78] Patel S.N., Abowd G.D., Reynolds M.S., Robertson T., Stuntebeck E. (2013). Sub Room Level Indoor Location System Using Wideband Power Line Positioning. U.S. Patent.

[79] Patel S.N., Truong K.N., Abowd G.D., Robertson T., Reynolds M.S. (2013). Sub-Room-Level Indoor Location System Using Power Line Positioning. U.S. Patent.

[80] Nishikawa K.i., Higashino T., Tsukamoto K. (2008). A new position detection method using leaky coaxial cable. IEICE Electron. Express.

[81] Weber M., Birkel U., Collmann R. Indoor RF Fingerprinting using leaky feeder cable considering environmental changes. Proceedings of the 6th International Conference on Mobile Technology, Application & Systems.

[82] See K.Y., So P.L., Kamarul A., Gunawan E. (2005). Radio-frequency common-mode noise propagation model for power-line cable. IEEE Trans. Power Deliv..

[83] Vukicevic A., Rubinstein M., Rachidi F., Bermúdez J.L. On the mechanisms of differential-mode to common-mode conversion in the broadband over power line (BPL) frequency band. Proceedings of the 2006 17th International Zurich Symposium on Electromagnetic Compatibility.

[84] Favre P., Candolfi C., Schneider M., Rubinstein M., Krahenbuehl P., Vukicevic A. Common mode current and radiations mechanisms in PLC networks. Proceedings of the 2007 IEEE International Symposium on Power Line Communications and Its Applications.

[85] Sugiura A., Kami Y. (2011). Generation and propagation of common-mode currents in a balanced two-conductor line. IEEE Trans. Electromagn. Compat..

[86] Adebisi B., Honary B. Comparisons of indoor PLC emissions measurement results and regulation standards. Proceedings of the 2006 IEEE International Symposium on Power Line Communications and its Applications.

[87] Potisk L., Hallon J., Orgon M., Fujdiak R. (2018). Electromagnetic compatibility of PLC adapters for in-home/domestic networks. J. Electr. Eng..

[88] Marthe E., Rachidi F., Ianoz M., Zweiacker P. Indoor radiated emission associated with power line communication systems. Proceedings of the 2001 IEEE EMC International Symposium. Symposium Record. International Symposium on Electromagnetic Compatibility (Cat. No. 01CH37161).

[89] Lopes P.A., Pinto J.M., Gerald J.B. (2012). Dealing with unknown impedance and impulsive noise in the power-line communications channel. IEEE Trans. Power Deliv..

[90] Vukicevic A., Rachidi F., Rubinstein M., Tkachenko S.V. (2006). On the evaluation of antenna-mode currents along transmission lines. IEEE Trans. Electromagn. Compat..

[91] Chariag D., Le Bunetel J., Khalil K., Raingeaud Y., Machmoum M., Guerin P. (2016). Analytical Modeling and Experimental Validation of Electromagnetic Field Radiated by In-house PLC Lines. J. Control Sci. Eng..

[92] Blankenbach J., Norrdine A., Hellmers H., Gasparian E. A novel magnetic indoor positioning system for indoor location services. Proceedings of the 8th International Symposium on Location-Based Services.

[93] Pirkl G., Lukowicz P. Robust, low cost indoor positioning using magnetic resonant coupling. Proceedings of the 2012 ACM Conference on Ubiquitous Computing.

[94] Sheinker A., Ginzburg B., Salomonski N., Frumkis L., Kaplan B.Z. (2012). Localization in 2D using beacons of low frequency magnetic field. IEEE J. Sel. Top. Appl. Earth Obs. Remote Sens..

[95] Sheinker A., Ginzburg B., Salomonski N., Frumkis L., Kaplan B.Z. (2013). Localization in 3-D using beacons of low frequency magnetic field. IEEE Trans. Instrum. Meas..

[96] Shirai R., Itoh Y., Hashimoto M. (2021). Make it Trackable: An Instant Magnetic Tracking System with Coil-Free Tiny Trackers. IEEE Access.

[97] Qian Y., Yan J., Guan H., Li J., Zhou X., Guo S., Jayakody D.N.K. (2017). Design of hybrid wireless and power line sensor networks with dual-interface relay in IoT. IEEE Internet Things J..

[98] Lai S.W., Messier G.G. The wireless/power-line diversity channel. Proceedings of the 2010 IEEE International Conference on Communications.

[99] Gheth W., Rabie K.M., Adebisi B., Ijaz M., Harris G., Alfitouri A. Hybrid power-line/wireless communication systems for indoor applications. Proceedings of the 2018 11th International Symposium on Communication Systems, Networks & Digital Signal Processing (CSNDSP).

[100] Zhu X., Zhu K., Heggo M. (2020). Hybrid Wireless-Power Line Communications for Indoor IoT Networks.

[101] Khan L.U. (2017). Visible light communication: Applications, architecture, standardization and research challenges. Digit. Commun. Netw..

[102] Pohlmann C. (2010). Visible light communication. Proceedings of the Seminar Kommunikationsstandards in der Medizintechnik.

[103] Lee C.G., Katz M. (2015). Visible light communication. Short-Range Wirel. Commun. Emerg. Technol. Appl..

[104] Ayub S., Kariyawasam S., Honary M., Honary B. A practical approach of VLC architecture for smart city. Proceedings of the IEEE 2013 Loughborough Antennas & Propagation Conference (LAPC).

[105] Ma X., Ding W., Yang F., Yang H., Song J. A positioning compatible multi-service transmission system based on the integration of VLC and PLC. Proceedings of the IEEE 2015 International Wireless Communications and Mobile Computing Conference (IWCMC).

[106] Gungor V.C., Sahin D., Kocak T., Ergut S., Buccella C., Cecati C., Hancke G.P. (2012). A survey on smart grid potential applications and communication requirements. IEEE Trans. Ind. Inform..

[107] Ma R., Chen H.H., Huang Y.R., Meng W. (2013). Smart grid communication: Its challenges and opportunities. IEEE Trans. Smart Grid.

[108] Bari A., Jiang J., Saad W., Jaekel A. (2014). Challenges in the smart grid applications: An overview. Int. J. Distrib. Sens. Netw..

[109] Bayindir R., Colak I., Fulli G., Demirtas K. (2016). Smart grid technologies and applications. Renew. Sustain. Energy Rev..

[110] Dileep G. (2020). A survey on smart grid technologies and applications. Renew. Energy.

[111] Liu K., Chen M., Cai E., Ma J., Liu S. (2018). Indoor localization strategy based on fault-tolerant area division for shipboard surveillance. Autom. Constr..

[112] Chen M., Liu K., Ma J., Gu Y., Dong Z., Liu C. (2019). SWIM: Speed-aware WiFi-based passive indoor localization for mobile ship environment. IEEE Trans. Mob. Comput..

[113] Ma Y., Liu K., Chen M., Ma J., Zeng X., Wang K., Liu C. (2020). ANT: Deadline-aware adaptive emergency navigation strategy for dynamic hazardous ship evacuation with wireless sensor networks. IEEE Access.

[114] Chen M., Ma J., Zeng X., Liu K., Chen M., Zheng K., Wang K. (2022). MD-Alarm: A Novel Manpower Detection Method for Ship Bridge Watchkeeping using WiFi Signals. IEEE Trans. Instrum. Meas..

[115] Lin Q., Son J. (2022). Sustainable Ship Management Post Covid-19 with In-Ship Positioning Services. Sustainability.

[116] Akinnikawe A., Butler-Purry K.L. Investigation of broadband over power line channel capacity of shipboard power system cables for ship communication networks. Proceedings of the 2009 IEEE Power & Energy Society General Meeting.

[117] Barmada S., Bellanti L., Raugi M., Tucci M. (2010). Analysis of power-line communication channels in ships. IEEE Trans. Veh. Technol..

[118] Antoniali M., Tonello A.M., Lenardon M., Qualizza A. Measurements and analysis of PLC channels in a cruise ship. Proceedings of the 2011 IEEE International Symposium on Power Line Communications and its Applications.

[119] Tsuzuki S., Yoshida M., Yamada Y., Murai K., Kawasaki H., Matsuyama K., Shinpo T., Saito Y., Takaoka S. Channel characteristic comparison of armored shipboard cable and unarmored one. Proceedings of the 2008 IEEE International Symposium on Power Line Communications and its Applications.

[120] Thrybom L., Neander J., Hansen E., Landernas K. (2015). Future challenges of positioning in underground mines. IFAC-PapersOnLine.

[121] Almada-Lobo F. (2015). The Industry 4.0 revolution and the future of Manufacturing Systems (MES). J. Innov. Manag..

[122] Henning K. (2013). Recommendations for Implementing the Strategic Initiative Industrie 4.0.

[123] Fernández-Caramés T.M., Fraga-Lamas P. (2018). A review on human-centered IoT-connected smart labels for the industry 4.0. IEEE Access.

[124] Ruppert T., Jaskó S., Holczinger T., Abonyi J. (2018). Enabling technologies for operator 4.0: A survey. Appl. Sci..

[125] Gladysz B., Santarek K., Lysiak C. (2017). Dynamic spaghetti diagrams. A case study of pilot RTLS implementation. International Conference on Intelligent Systems in Production Engineering and Maintenance.

[126] Blum M., Schuh G. Towards a Data-oriented Optimization of Manufacturing Processes. Proceedings of the 19th International Conference on Enterprise Information Systems.

[127] Kwon S.G., Kwon O.J., Kwon K.R., Lee S.H. (2021). UWB and MEMS IMU Integrated Positioning Algorithm for a Work-Tool Tracking System. Appl. Sci..

[128] Villalpando-Hernandez R., Muñoz-Rodríguez D., Vargas-Rosales C. (2021). Relational Positioning Method for 2D and 3D Ad Hoc Sensor Networks in Industry 4.0. Appl. Sci..

[129] Cheng C.H., Kuo Y.H., Lam H., Petering M. (2021). Real-Time Location-Positioning Technologies for Managing Cart Operations at a Distribution Facility. Appl. Sci..

[130] Li Y., Zhuang Y., Lan H., Zhou Q., Niu X., El-Sheimy N. (2015). A hybrid WiFi/magnetic matching/PDR approach for indoor navigation with smartphone sensors. IEEE Commun. Lett..

[131] Li Y., Zahran S., Zhuang Y., Gao Z., Luo Y., He Z., Pei L., Chen R., El-Sheimy N. (2019). IMU/magnetometer/barometer/mass-flow sensor integrated indoor quadrotor UAV localization with robust velocity updates. Remote Sens..

[132] Guo X., Ansari N., Hu F., Shao Y., Elikplim N.R., Li L. (2019). A survey on fusion-based indoor positioning. IEEE Commun. Surv. Tutor..

[133] Gotz M., Rapp M., Dostert K. (2004). Power line channel characteristics and their effect on communication system design. IEEE Commun. Mag..

[134] Sancha S., Cañete F., Díez L., Entrambasaguas J. A channel simulator for indoor power-line communications. Proceedings of the 2007 IEEE International Symposium on Power Line Communications and its Applications.

[135] Cañete F.J., Cortés J.A., Díez L., Entrambasaguas J.T. (2011). A channel model proposal for indoor power line communications. IEEE Commun. Mag..

[136] Biglieri E. (2003). Coding and modulation for a horrible channel. IEEE Commun. Mag..

[137] Latchman H.A., Katar S., Yonge L., Gavette S. (2013). Homeplug AV and IEEE 1901: A Handbook for PLC Designers and Users.

[138] (2010). IEEE Standard for Broadband over Power Line Networks: Medium Access Control and Physical Layer Specifications.

[139] (2018). IEEE Standard for Medium Frequency (Less than 12 MHz) Power Line Communications for Smart Grid Applications.

[140] (2013). IEEE Standard for Low-Frequency (Less than 500 kHz) Narrowband Power Line Communications for Smart Grid Applications.

[141] ITU Telecommunication Standardization Sector, S.G.T.S., Media, D.S, Networks (2010). ITU-T G.9960: Access networks. Premises Networks Unified High-Speed Wire-Line Based Home Networking Transceivers.

[142] Wi-SUN Alliance (2014). HomePlug AV Specification Version 2.1. https://docbox.etsi.org/Reference/homeplug_av21/homeplug_av21_specification_final_public.pdf.

[143] Cruz-Roldán F., Pinto-Benel F.A., Osés del Campo J.D., Blanco-Velasco M. (2016). A wavelet OFDM receiver for baseband power line communications. J. Frankl. Inst..

[144] Pinto-Benel F.A., Cruz-Roldán F. (2016). A bandpass wavelet OFDM system for power line communications. J. Frankl. Inst..

